# Modulation of the molecular spintronic properties of adsorbed copper corroles

**DOI:** 10.1038/ncomms8547

**Published:** 2015-06-26

**Authors:** Fan Wu, Jie Liu, Puneet Mishra, Tadahiro Komeda, John Mack, Yi Chang, Nagao Kobayashi, Zhen Shen

**Affiliations:** 1 State Key Laboratory of Coordination Chemistry, Collaborative Innovation Center of Advanced Microstructures, Collaborative Innovation Center of Chemistry for Life Sciences, School of Chemistry and Chemical Engineering, Nanjing University, Nanjing 210093, China; 2 Institute of Multidisciplinary Research for Advanced Materials (IMRAM, Tagen), Tohoku University, 2-1-1, Katahira, Aoba-Ku, Sendai 980-0877, Japan; 3 JST, CREST, 4-1-8 Honcho, Kawaguchi, Saitama 332-0012, Japan; 4 Department of Chemistry, Rhodes University, Grahamstown 6140, South Africa; 5 Department of Chemistry, Graduate School of Science, Tohoku University, Aobayama, Sendai 980-8578, Japan

## Abstract

The ability to modulate the spin states of adsorbed molecules is in high demand for molecular spintronics applications. Here, we demonstrate that the spin state of a corrole complex can be tuned by expanding its fused ring as a result of the modification to the d–π interaction between the metal and ligand. A bicyclo[2.2.2]octadiene-fused copper corrole can readily be converted into a tetrabenzocorrole radical on an Au(111) substrate during the sublimation process. In the scanning tunnelling spectroscopy spectrum, a sharp Kondo resonance appears near the Fermi level on the corrole ligand of the tetrabenzocorrole molecule. In contrast, a non-fused-ring-expanded copper corrole molecule, copper 5,10,15-triphenylcorrole, shows no such Kondo feature. Mapping of the Kondo resonance demonstrates that the spin distribution of the tetrabenzocorrole molecule can be further modified by the rotation of the *meso*-aryl groups, in a manner that could lead to applications in molecular spintronics.

## Abstract (web summary)

Modulation of the spin states of molecules adsorbed on metal surfaces facilitates potential spintronic applications. Here, the authors demonstrate the tuning of the electronic interaction between a gold surface and the ligand of a corrole complex, tuning its spin state through fused-ring-expansion.

## Graphical abstract

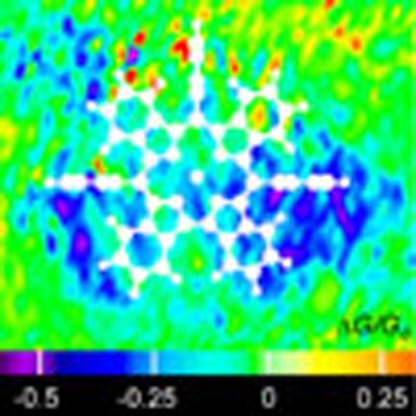

Molecular spintronics is an emerging research field, in which organic molecules are placed between electrodes, and the electron conductance is controlled by the free circulation of the electron spin of a single molecule^1^,^2^,^3^. The modulation of conductance through molecules in this manner has been demonstrated by molecular-level scanning tunnelling microscopy (STM) measurements of Kondo resonance signals, which are associated with the exchange coupling between the unpaired spins of the paramagnetic molecules and the conduction band electrons of the metal substrate. For example, these signals have been observed on metal surfaces for several metallophthalocyanines^4^,^5^,^6^ and metalloporphyrins^7^,^8^,^9^. The unpaired π-orbitals of metal complexes tend to play an important role in spin-sensitive electron transfer, because π-radical orbitals are delocalized and can be coupled more efficiently with the conduction band^10^,^11^. Recent advances in the rational modulation of the energies of the frontier molecular orbitals (MOs) of the macrocycle ligands^12^,^13^,^14^,^15^ offer the prospect of controlling charge and spin at the molecular level in a manner that could facilitate molecular spintronics applications.

Corroles are porphyrin analogues with a direct pyrrole–pyrrole link, which can stabilize higher oxidation states of the coordinated transition metal ions^16^,^17^. In recent decades, there has been extensive research on corrole complexes, since the ligands may have a non-innocent character in which a one-electron dianionic radical rather than the normal closed-shell trianion binds to the central metal^18^. Copper corroles stand out as the most notable in this regard^18^,^19^,^20^,^21^. It has been demonstrated that considerable electron density can flow into the copper 3*d*_*x*^2^–*y*^2^_ orbital from the highest occupied molecular orbital (HOMO) of the corrole ligand π-system; this specific *d*–π interaction can lead to a saddling distortion that is observed in both X-ray crystal and density functional theory (DFT)-optimized structures^20^. Since the spin states of copper corroles are determined by the *d*–π interaction, special attention has been paid to how the spin-state properties can be modulated by modifying the corrole ligand. In this study, it is demonstrated that a triplet ground state can be switched on in a manner that may be suitable for spintronics by introducing fused benzene rings on the corrole periphery, and that the spin properties are further modulated on the Au(III) substrate by a rotation of the *meso*-aryl groups. A bicyclo[2.2.2]octadiene (BCOD)-fused copper corrole (Cu-BCOD) has been prepared, which can readily be converted into a tetrabenzocorrole (Cu-Benzo) in quantitative yield by heating *in vacuo*. A copper 5,10,15-triphenylcorrole (Cu-TPC) has also been prepared so that the effect of fused-ring-expansion on the Kondo resonance signals can be readily analysed.

## Results

### Preparation and characterization of the adsorbed molecules

**Figure 1:**
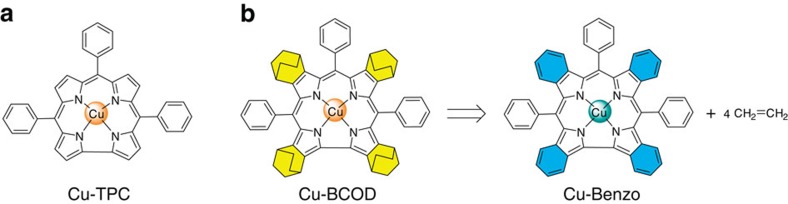
Schematic illustration of Cu corroles used in our study. (**a**) Structure formula of Cu-TPC. The orange ball represents the central Cu(III) ion. (**b**) Cu-BCOD was converted into Cu-Benzo via a retro-Diels–Alder reaction during a sublimation process with the extrusion of ethylene molecules. The dark-green ball represents the central Cu(II) ion. The BCOD and Benzo moieties are highlighted in yellow and blue, respectively.

The Cu-BCOD sample (Fig. 1b) was newly synthesized using 4,7-dihydro-4,7-ethano-2*H-*isoindole as the starting material (see Methods), and Cu-TPC (Fig. 1a) was synthesized according to the previously reported procedures^22^. The molecule was transferred to a Au(111) substrate using a sublimation method under ultra-high vacuum conditions, by heating the sample in a Ta boat at ∼300 °C (refs 23, 24). During the sublimation process, Cu-BCOD was converted into the Cu-Benzo molecule via a retro-Diels–Alder reaction involving the extrusion of four ethylene molecules from the fused BCOD rings as shown in Fig. 1b. This was confirmed by time-of-flight (TOF) secondary ion mass spectrometry (MS) (Supplementary Fig. 1). In contrast, the sublimation of Cu-TPC resulted in no molecular decomposition.

**Figure 2:**
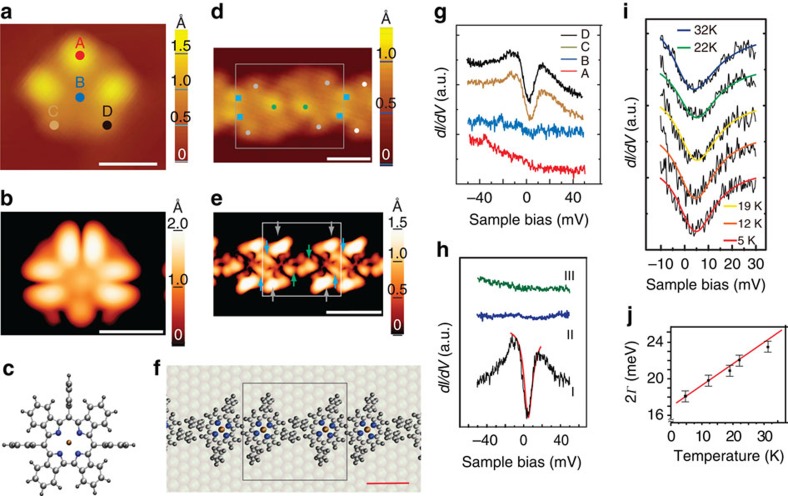
STM image and Kondo resonance of Cu-TPC and Cu-Benzo. (**a**) STM topographic image, (**b**) simulated STM image and (**c**) optimized structural model of Cu-Benzo monomer on Au(111). Corresponding tunnelling conditions of *V*_sample_=−0.8 V and *I*_tunnel_=0.3 nA. Scale bars, 10 Å and colour scales indicate height information. In **c**, large (small) grey spheres represent C (H) atoms, while blue and gold spheres correspond to N and Cu atoms, respectively. (**d**–**f**) Same as **a**–**c** but for a Cu-TPC chain on Au(111) surface. The box indicates the unit cell that appears periodically in the chain. Prominent features are marked by dots in **d**, whose corresponding protrusions in the simulation are shown by arrows with the same colour. The colour scheme of atoms in **f** is same as that in **c**. (**g**) d*I/*d*V* spectra obtained for Cu-Benzo monomer at positions A–D in **a**. (**h**) Comparison of the d*I/*d*V* spectra at the ligand positions of Cu-Benzo on Au(111) (I, black), on Cu(111) (II, blue) and Cu-TPC on Au(111) (III, green). Red curve in **i** shows the result of the Fano fitting. (**i**) Temperature dependence of the Fano dip of Cu-Benzo measured in the temperature region of 4.7–32 K. (**j**) Width of the dip at half maximum (**2***Γ*) versus temperature for the Kondo dip near the Fermi level. The solid curve indicates the fitted curve. The error bars were estimated by measuring the scattering of the data in the heat cycles repeated eight times.

An STM image of an isolated Cu-Benzo molecule adsorbed on Au(111) (Fig. 2a) exhibits three characteristic protruded areas together with a square-like region. The STM image simulations were calculated for a sample bias voltage of −0.8 V (Fig. 2b), using a Vienna Ab initio Simulation Package (VASP)-optimized molecular structure (Fig. 2c). The simulation image contains three protruded spots similar to those observed in Fig. 2a. The distance between the two bright spots in Fig. 2a is ∼13.0 Å, which is close to the separation of the centres of the *meso*-aryl rings of 12.6 Å shown in Fig. 2c. In the rest of the molecule, a square-like protruded area can be identified, which is similar to that observed in the STM image. Each protruded spot has a node in the middle. However, the node is not visible in the observed STM image. This discrepancy may be related to a tilting of the phenyl rings upon adsorption, which will be discussed in greater detail below. The aryl ring marked A in Fig. 2a will be referred to as the *y*-axis *meso*-aryl rings, while the other two will be referred to as the *x*-axis *meso*-aryl rings.

In contrast, Cu-TPC molecules form a chain on the Au(111) surface, similar to that reported in hydrocarbon π radicals^25^. In the unit cell marked by the white square, two Cu-TPC molecules rotated by 180° with respect to each other (see Fig. 2d). The optimized model structure is shown in Fig. 2f together with a simulated STM image for a bias voltage of −0.8 V (Fig. 2e), which indicates that three *meso*-aryl rings appear protruded as is also observed for Cu-Benzo molecules.

### Kondo resonance and spintronics properties

The spin states of Cu-Benzo and Cu-TPC molecules were investigated by detecting the Kondo resonance using STM. The Kondo effect is caused by the interaction between the conduction band electrons and localized spins^26^. Recently, it has been demonstrated that Kondo resonance can be formed by the spin of a delocalized molecular π-radical and the conduction electrons of the substrate^27^,^28^,^29^,^30^. The Kondo resonance appears in the scanning tunnelling spectroscopy (STS) spectrum near the Fermi level either as a sharp peak or dip, which is determined by the Fano resonance effect-type interference between the tunnelling electron^31^. The STS spectra obtained for Cu-Benzo molecule at the A–D positions (Fig. 2a) are provided in Fig. 2g. At positions A and B, on the *y*-axis *meso*-aryl ring and the central Cu atom, no STS features are observed. In contrast, spectra obtained at positions C and D of the corrole ligand show signals at the Fermi level, whose narrow width and shape are consistent with a Kondo dip feature and are similar to those that have been reported for Cu porphyrin molecules^32^.

The Kondo dip was analysed using the following functions, which are often used for Fano shape resonance^33^,^34^,^35^: 
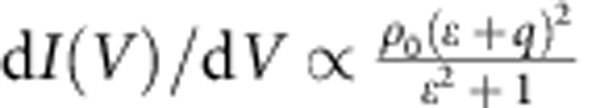
 and 
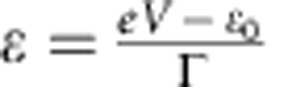
 where *q* is the Fano parameter, *ɛ*_*0*_ is the peak position and *Γ* is the half-width of the peak. The peak-width change with sample temperature (see Fig. 2i,j) was examined to prove that the zero-bias peak originates from Kondo resonance. The peaks are fitted with the Fano functions, the result of which is shown as solid curves in Fig. 2j. At elevated temperatures, the measurement of the Kondo resonance was hampered due to the thermal diffusion of the molecule, because of weak bonding between the Cu-Benzo molecule and the Au(111) surface. Thus, the temperature range shown in Fig. 2i,j is narrow. Nevertheless, an examination of the peak width shows clear variation with sample temperatures. Nagaoka *et al.*^35^ introduced a formula for the Kondo peak-width variation with temperature (*Γ*(*T*)) using the Fermi liquid theory: 
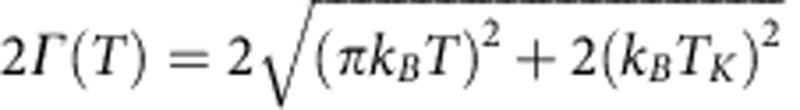
 where *k*_B_ is the Boltzmann constant and *T*_K_ is the Kondo temperature. The observed data (solid circles) were successfully fitted using this formula. The fitted curve is shown in Fig. 2j as a solid line, which gives *T*_K_∼105 K. This is consistent with the assignment of the zero-bias peak to Kondo resonance.

The STS spectrum measured for Cu-TPC exhibits no Kondo feature at the ligand position (III of Fig. 2h). This indicates the absence of molecular spin and provides direct spectroscopic evidence for the singlet ground state. When electron paramagnetic resonance (EPR) spectra were measured, Cu-TPC and Cu-BCOD were found to be EPR silent, as would be anticipated for a Cu(III) singlet ground state, while the EPR spectrum of Cu-Benzo in frozen CHCl_3_ exhibits a highly distinctive Cu(II) dimer signal (Supplementary Fig. 2)^36^,^37^.

## Discussion

To examine electronic and spin states more precisely, geometry optimizations and calculation of electronic states were carried out for singlet and triplet ground states of Cu-TPC, Cu-BCOD and Cu-Benzo using the hybrid B3LYP functional with 6–31G(d) basis sets. As anticipated, the singlet ground state was predicted to be more stable for Cu-TPC and Cu-BCOD (by 0.4 and 2.6 kcal mol^−1^, respectively), but not for Cu-Benzo (by 5.1 kcal mol^−1^). For all three complexes, the geometry of the singlet ground state is predicted to be significantly more saddled than the triplet states in a manner that is consistent with Kahn's concept of orthogonal magnetic orbitals^38^. The concept of orthogonal orbitals can be readily applied to rationalize the magnetic coupling of metal porphyrin π–cation radical complexes^39^,^40^. The theory is generally based on the symmetry of the orbitals on the metal and the ligand that contain unpaired electrons. In a planar structure, the metal orbital is strictly orthogonal to the ligand π-radical orbital and this results in an *S*=1 triplet state. On the other hand, when the orbitals of the metal and ligand are not strictly forbidden by symmetry and hence partially overlap, as in a saddled structure, there is antiferromagnetic coupling resulting in an *S*=0 singlet state, which cannot be distinguished from dative bond formation. This concept can be applied to metallocorroles as well due to the similar frontier π-MOs in shape^18^. Almost all of the crystal structures that have been reported for copper corroles have saddled conformations due to the *d*–π interaction^41^. For Cu-Benzo, however, a planar conformation is observed in the crystal structure (see Supplementary Fig. 3 and Supplementary Data 1)^42^.

**Figure 3:**
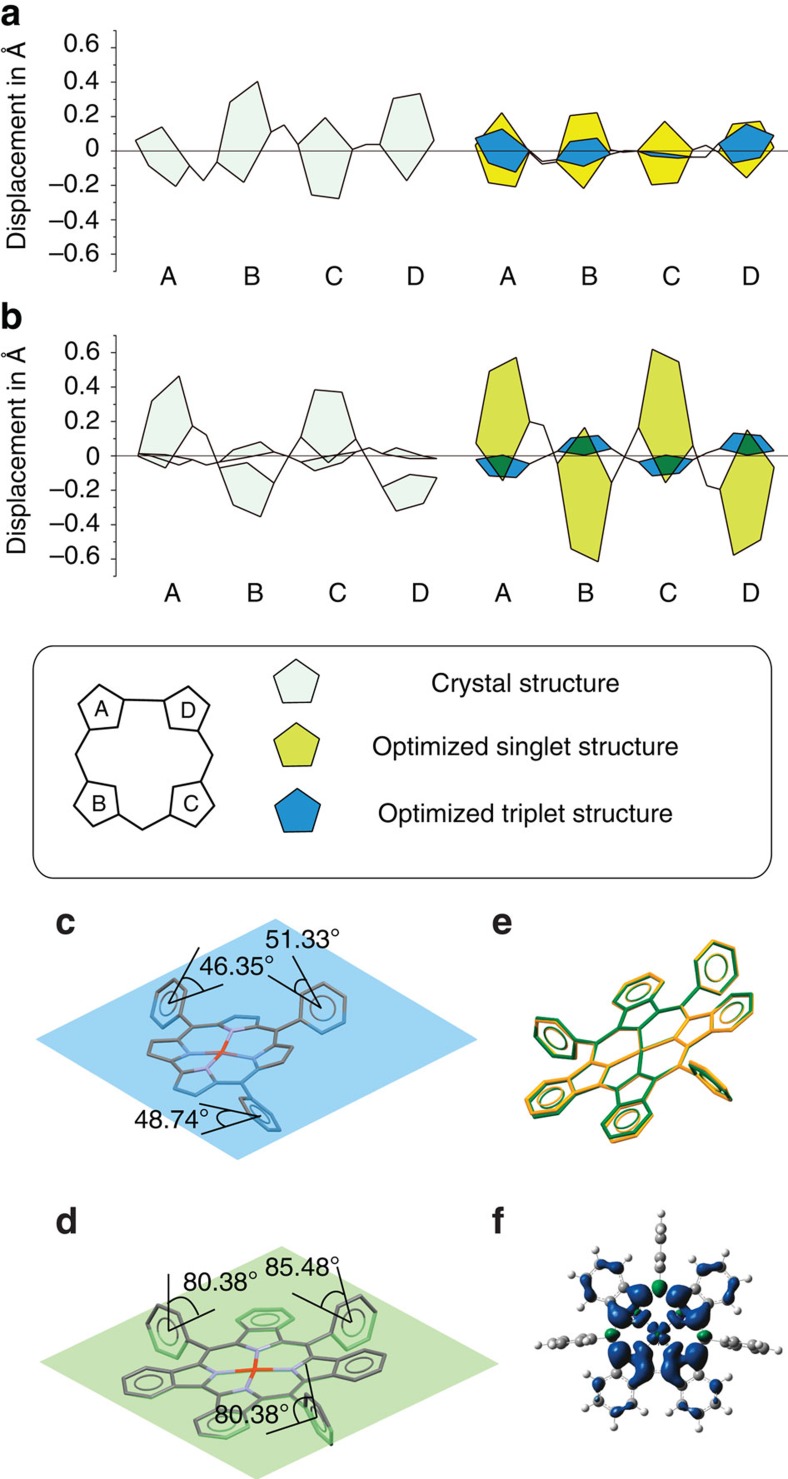
Comparison and analysis of the crystal and optimized structures. (**a**) The distortions of Cu-TPC and (**b**) Cu-Benzo in the crystal and B3LYP-optimized structures are compared. The displacements from the mean plane of the 23 ligand atoms are illustrated in clothes-line diagrams. The four pyrrole moieties are labelled as A, B, C and D. The coloured pyrrole moieties represent crystal structure (grey), optimized singlet structure (yellow) and optimized singlet structure (blue). The dihedral angles between *meso*-aryl groups and 23 ligand atoms' least square planes (the coloured plane) in the crystal structures of Cu-TPC (**c**) and planar Cu-Benzo (**d**). (**e**) Overlay of planar crystal (green) and B3LYP-optimized triplet structure (yellow) of Cu-Benzo. (**f**) Spin density plot of Cu-Benzo for the *S*=1 triplet state (isospin=0.001), calculated with B3LYP/6–31G(d) level of theory.

When the B3LYP-optimized structures are compared with the crystal structures by the displacements of the 23 core ligand atoms (see Supplementary Fig. 4, Supplementary Note 1 and Supplementary Data 2), the saddling conformation of the Cu-TPC macrocycle can be clearly observed in the edge-on view, similar to the optimized structure for the singlet ground state as shown in Fig. 3a. Interestingly, the asymmetric unit cell of Cu-Benzo contains two molecules with different conformations. One has a saddled structure, which also closely matches the optimized singlet structure (Fig. 3b), whereas the other one adopts a planar conformation that overlaps perfectly with the optimized triplet structure. The crystal and optimized structures of the planar conformation of Cu-Benzo are essentially identical, including that of the three *meso*-aryl groups (Fig. 3e). The *meso*-aryl groups of Cu-Benzo are almost perpendicular to the mean corrole plane with an average dihedral angle of 82.9° (Fig. 3d), while the dihedral angle for Cu-TPC is only 48.8° (Fig. 3c). The spin density plots for the planar triplet state (Fig. 3f) demonstrate that there is ferromagnetic coupling between the copper 3*d*_*x*^2^–*y*^2^_ orbital and the benzocorrole π-orbital. There is almost no spin density on the *meso*-aryl groups, since they lie orthogonal to the corrole plane.

Copper corroles represent an unusual exception among metallocorroles, because saddled structures have been a shared feature of all of the complexes reported to date, even in the absence of steric crowding at the ligand periphery. It has been widely accepted that copper corroles are saddled, since there is an energetically favourable 3*d*_*x*^2^–*y*^2^_ and ligand π–HOMO interaction. Upon fused-ring-expansion to form Cu-Benzo, however, there are significant changes in the relative energies of the frontier MOs in a manner that discourage overlap between the 3*d*_*x*^2^–*y*^2^_ orbital of the metal ion and the occupied frontier π-orbital, which has large MO coefficients on the pyrrole nitrogens, and this favours a planar conformation. Since only non-planar conformations have been reported previously in the crystal structures of tetraphenyltetrabenzoporphyrins^43^,^44^, the planar structure of Cu-Benzo is unique in this regard.

**Figure 4:**
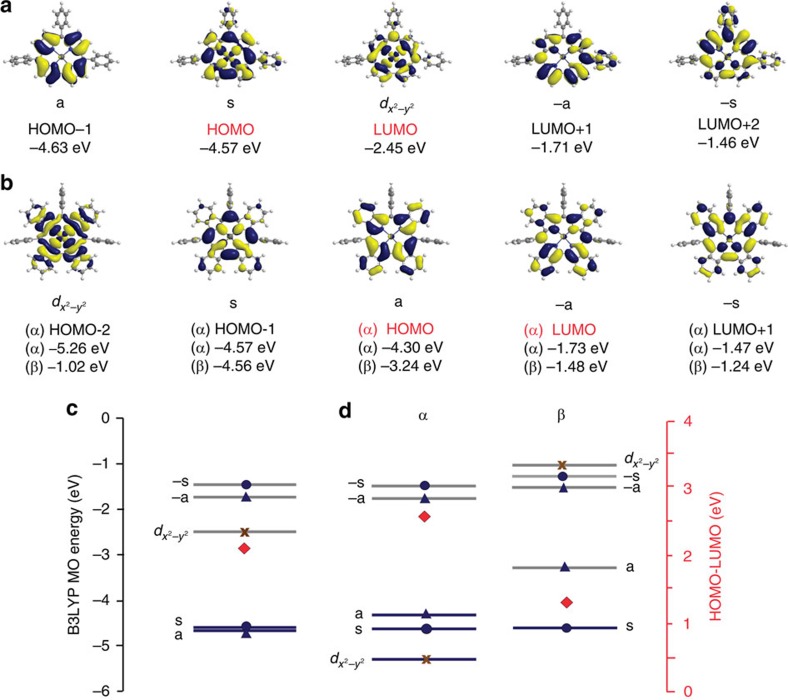
Contour plots and energy levels of the B3LYP-optimized structures. (**a**,**b**) Frontier MO contour plots and corresponding energy values of Cu-TPC (**a**) and Cu-Benzo (**b**). Michl's **a**, **s**, **−a** and **−s** nomenclature is used to describe the frontier π-MOs with *M*_L_=±4 and ±5 nodal patterns. (**c**,**d**) Schematic energy diagram of the frontier MOs of Cu-TPC (**c**) and Cu-Benzo (**d**). Occupied and empty MOs are highlighted with blue and grey lines, respectively, and blue circles, triangles and brown crosses are used to denote the **s** and **−s** MOs, **a** and **−a** MOs and the *d*_*x*^2^–*y*^2^_ MOs, respectively. The predicted HOMO–LUMO gaps are denoted with red diamonds and are plotted against a secondary axis.

As has been reported previously, the frontier π-MOs of corroles are very similar to the a_1u_, a_2u_ and e_g_ frontier π-MOs of porphyrins, despite the loss of one *meso*-carbon atom on the inner ligand perimeter. Thus a perimeter-model approach can be adopted to study trends in their energies^45^. Michl referred to the two frontier MOs derived from the HOMO and lowest unoccupied molecular orbital (LUMO) of the parent perimeter in which angular nodal planes lie on the *y*-axis as the **a** (a_1u_) and −**a** (e_g*y*_) MOs, while those which lie on antinodes are referred to as the **s** (a_2u_) and −**s** (e_g*x*_) MOs^45^,^46^,^47^,^48^. For Cu-TPC, saddling enables the **s** MO with large MO coefficients on the pyrrole nitrogens to mix significantly with the 3*d*_*x*^2^–*y*^2^_ orbital of the central metal, so that the electron spins are paired. This overlap can be seen in the angular nodal patterns of the HOMO and LUMO of Cu-TPC in Fig. 4a. The frontier MOs of Cu-BCOD should be similar to those of Cu-TPC (Supplementary Fig. 5) because, as has been reported previously in the context of BCOD-fused [14] triphyrin(2.1.1) compounds^49^, the presence of the BCOD moieties has only a minor effect on the π-conjugation system.

The electronic structure of Cu-Benzo (Fig. 4b,d) is predicted to be markedly different from that of Cu-TPC (Fig. 4a,c), since the triplet state is predicted to be more stable than the corresponding singlet state. The α-spin **a** MO is the singly occupied molecular orbital (SOMO), since its energy is higher than that of the α- and β-spin **s** MO (Fig. 4d). This can be attributed to a destabilization of the energy of **a** MO due to the effect of fused-ring-expansion on the π-system. A significant destabilizing antibonding interaction has been reported previously at the point of fused-ring-attachment on the β-pyrrole carbons for other benzene-fused porphyrinoids^50^,^51^,^52^. The β-spin **a** MO is unoccupied and hence is the LUMO of Cu-Benzo (Fig. 4d). The macrocycle is oxidized to form a corrolate π-cation radical with an unpaired spin and a central Cu(II) ion.

The theoretical considerations disscussed above can account for the experimental observation of the Kondo resonance. For the Cu-TPC molecule, it is predicted that the ligand π-orbital is paired and no Kondo resonance is formed, which agrees with the absence of the Kondo feature for this molecule. For the Cu-Benzo molecule, a ground state is calculated, which is consistent with the observation of the Kondo resonance measured at the corrole ligand position. The SOMO level can be attributed to the spin impurity of Kondo resonance. It could be argued that the partial filling of the SOMO level may be due to charge transfer from the Au(111) substrate as is the case with Cu-phthalocyanine (Pc) and NiPc on Ag(111)^6^. However, as shown in plot II of Fig. 2h, the Kondo resonance is absent when the molecule is adsorbed on a more reactive Cu substrate where significantly more charge transfer is expected. This possibility can, therefore, be eliminated from consideration. It is noteworthy that the spin expected at the Cu position for the triplet state is not detected. This may be due to the low *T*_*K*_ for the Kondo state formed by the Cu atom, which is too low to be observed at ∼4 K. Kondo resonance has been reported to be absent previously for a CuPc molecule for similar reasons^6^.

**Figure 5:**
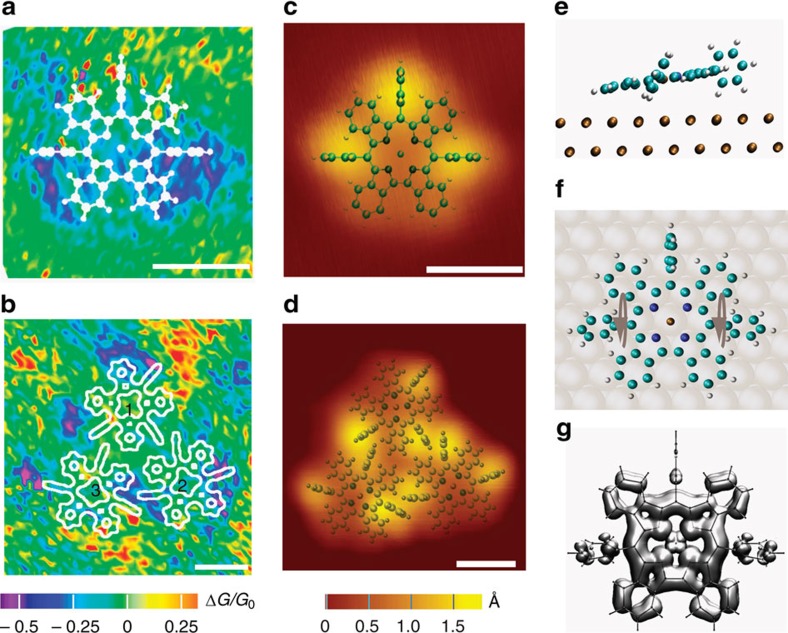
Kondo peak mapping to detect spin distribution. Kondo resonance mapping for (**a**) Cu-Benzo monomer and (**b**) trimer, where molecule models are superimposed by white lines. Kondo mapping is obtained by measuring d*I/*d*V* curve in the vicinity of the Fermi level at the lattice points of a 64 × 64-grid and expressed as normalized conductance change of Δ*G*/*G*_0_, where Δ*G* and *G*_0_ correspond to the conductance change and background conductance at the Fermi level, respectively. Colour map is shown at the bottom of **b**. Corresponding topographic images for the monomer and trimer are shown in **c** and **d**, respectively, with structural model of the molecules. Scale bars, 10 Å (**a**–**d**). (**e**,**f**) Side view (**e**) and top view (**f**) of titled Cu-Benzo on Au(111) with rotation of the two *meso*-aryl groups that are aligned with the *x*-axis followed by tilting of the molecule. Spheres of white, light-blue, dark-blue and bronze correspond to H, C, N and Cu, respectively. Au atoms are in brown in **e** but large grey in **f**. The arrows in **f** illustrate the rotation of the *meso*-aryls. (**g**) Calculated SOMO distribution for the models in **e** and **f**. Note that there is density at the *x*-axis *meso*-aryls, which is absent in the **a** MO of Cu-Benzo.

It is possible to examine whether the distribution of the SOMO level coincides with the positions at which the Kondo resonance is detected by Kondo mapping (see Methods). Figure 5 contains the Kondo mapping of the monomer and trimer Cu-Benzo molecules together with their topographic images. The mapping is obtained by measuring the d*I/*d*V* curve in the vicinity of the Fermi level at the lattice points of a 64 × 64-grid. In the mapping image, the Kondo resonance is observed at the ligand position, but is missing at the Cu position. The stronger Kondo resonance signal mainly lies near the peripheral fused benzo rings and the two *x*-axis *meso*-aryl rings. For the trimer case shown in Fig. 5b, molecules 1 and 2 follow the same trends as the monomer. However, there are missing Kondo resonances at the opposite side of the *y*-axis *meso*-aryl ring in molecule 3. There are two obvious discrepancies between the distribution of the SOMO and the measured Kondo mapping image. First, the Kondo resonance is observed at the two *x*-axis *meso*-aryl rings but there is little spin density at these aryl positions in the calculated SOMO distribution, and, secondly, the strong Kondo resonance that appears only on the opposite side of the central *y*-axis *meso*-aryl ring.

The obvious explanation for this is a change in the conformation when the molecules are adsorbed on Au(111). To gain van der Waals force, the corrole perimeter on the opposite side of the *y*-axis *meso*-aryl ring approaches the surface. This can best be realized through a rotation around the C–C bond, which connects the aryl ring to the *meso*-position of the corrole. To model this, the structure of Cu-Benzo was optimized by DFT on Au(111) using the VASP code. The results are shown in Fig. 5e,f, in which the two *x*-axis *meso*-aryls are rotated by ∼45° with respect to the corrole plane, and as a result, the molecule is tilted (see Supplementary Fig. 6 and Supplementary Note 2 for the relation with the STM image). Figure 5g contains the isosurface of the SOMO for the rotated *meso*-aryl ring conformation. Electron density is observed at the *x*-axis *meso*-aryls in contrast with what is predicted for conformations with perpendicular *meso*-aryl rings. This would account for the distribution of the Kondo resonance at the aryl positions and provides another clear demonstration of spin control by tuning of the ligand conformation, since the rotation of the *meso*-aryls changes the distribution of the molecular spins.

The tilted configuration of the adsorption also accounts for the strong Kondo resonance on the opposite side of the central *y*-axis *meso*-aryl ring. The Kondo resonance can be modulated even within a single SOMO by introducing variations in the height from the surface at each point in the orbital. As can be seen in Fig. 5e, the height of the SOMO from the substrate is higher at the opposite side of the central aryl ring, which should cause a variation in the interaction between the spin impurity and the substrate. This results in a spatial variation in both the *T*_*K*_ value and the magnitude of the Kondo resonance. At positions closer to the substrate, both the *T*_*K*_ value and the magnitude increases. A similar phenomenon has been reported previously by the Sendai group for a double-decker TbPc_2_ molecule. In this context, an unpaired π-orbital of the upper Pc ligand works as the spin centre for the Kondo resonance^28^. The upper Pc ligand tends to be tilted, which makes the height of each position of the SOMO from the substrate unequal.

In summary, fused-ring-expansion of the corrole ligand has been demonstrated to result in a unique spin state, in a manner that could lead to applications in molecular spintronics. When the Cu-BCOD molecules were sublimed onto the Au (111) surface from a heated Ta boat, they were converted into Cu-Benzo molecules in quantitative yield by a retro-Diels–Alder reaction. The destabilization of the **a** MO of Cu-Benzo results in a planar structure and the oxidation of the ligand leads to the formation of an uncoupled spin and hence a triplet ground state. The use of the sublimation method rather than a drop-cast method to form the Cu-Benzo molecules on the metal surface eliminates the possibility of the dimer formation that is observed by EPR spectroscopy in solution, which would remove the scope for molecular spintronics. This approach may prove to be useful in building molecular circuits. When the spin state of the corrole ligand of Cu-Benzo was characterized by STS, a Kondo resonance peak was observed as a sharp feature in the d*I/*d*V* curve at the Fermi level. In contrast, no Kondo features were observed for Cu-TPC. The sensitivity of the spin behaviour to fused-ring-expansion was analysed with DFT calculations, and the experimental data were found to be consistent with the predicted trends in the energies of the frontier MOs. Mapping of the Kondo resonance demonstrates that the spin distribution of the Cu-Benzo molecule can be modified by the rotation of the *meso*-aryl groups, in a manner that could lead to applications in molecular spintronics.

## Methods

### Synthesis of Cu corroles

Cu-TPC was synthesized according to the previously reported procedures^22^. 4,7-Dihydro-4,7-ethano-2H-isoindole was synthesized following literature procedures^53^. 4,7-Dihydro-4,7-ethano-2H-isoindole (284 mg, 2 mmol) and benzaldehyde (102 μl, 1 mmol) were dissolved in 80 ml of a 1:1 (v/v) solvent mixture of H_2_O and CH_3_OH mixture. HCl (36%, 1 ml) was then added, and the reaction was stirred at room temperature for 3 h. The mixture was extracted with CHCl_3_, washed with water twice and dried with Na_2_SO_4_. The CHCl_3_ solution was diluted to 60 ml. Tetrachloro-*p*-benzoquinone (246 mg, 1 mmol) and copper(II) acetate (80 mg, 0.4 mmol) were added and the mixture was refluxed for 1 h. The reaction mixture was washed with a saturated solution of NaHCO_3_ and then the organic solvent was removed under reduced pressure. The residue was purified on silica gel chromatography using (1:2, v/v) CH_2_Cl_2_/*n*-hexane as eluents, and the first yellow fraction was collected. Subsequent recrystallization from CH_2_Cl_2_/CH_3_OH afforded pure Cu-BCOD (74 mg, 19%) as a mixture of diastereomers. Cu-BCOD was heated to 250 °C under vacuum (2 mm Hg) for 20 min to afford Cu-Benzo in quantitative yield.

### Chemical characterization

Ultraviolet–visible (Vis) absorption spectra (Supplementary Fig. 7) was recorded with a SHIMADZU UV-2550 spectrometer. Matrix-assisted laser desorption/ionization (MALDI)-TOF MS data (Supplementary Figs 8 and 9) were measured on Bruker Daltonics autoflex^II^ and AB SCIEX 4800 Plus spectrometers. ^1^H NMR and ^1^H^1^H-COSY NMR spectra (Supplementary Figs 10 and 11) were recorded on a Bruker DRX500 spectrometer. Cu-BCOD: ^1^H NMR (500 MHz, CDCl_3_): *δ*7.76–7.42 (m, 15H), 6.62 (br s, 2H), 6.35 (m, 2H), 6.10 (br s, 4H), 4.45 (br s, 2H), 3.06 (m, 2H), 2.51 (br s, 2H), 2.38 (br s, 2H), 1.71–0.94 (m, 16H); ultraviolet/Vis: *λ*_max_ 412 nm; high-resolution-MS (MALDI-TOF): (*m*/*z*) [M]^+^ calcd. for C_61_H_47_CuN_4_: 898.3091, found: 898.3093. Cu-Benzo: ultraviolet/Vis: *λ*_max_ 456 nm; high-resolution-MS (MALDI-TOF): (*m*/*z*) [M]^+^ calcd. for C_53_H_31_CuN_4_: 786.1840, found: 786.1839.

### TOF-SIMS analysis of Cu-Benzo

TOF secondary ion MS (TOF-SIMS) measurements were performed using an ION-TOF GmbH (Münster, Germany) instrument equipped with a pulsed liquid metal cluster bismuth (Bi^3+^) primary ion gun (energy 25 kV). The instrument was used in the static mode, with a beam of primary ions of <1,012–1,013 ions per cm^2^ and an ion current of ∼0.4 pA. First, the Au(111)/mica substrate was prepared by annealing with a hydrogen torch to eliminate surface contamination. The Au(111) surface was introduced into a vacuum chamber and Cu-BCOD molecules (chemical formula C_61_H_47_CuN_4_, with a molecular of ∼889 a.u.) were sublimated onto the Au(111) surface from a Ta boat heated at ∼350 °C. The sample was transferred in the air to the TOF-SIMS chamber. The results demonstrated that the major molecule on the surface was Cu-Benzo (C_53_H_31_N_4_Cu, mass ∼786 a.u.), since four ethylene molecules were detached during the sublimation process.

### STM and STS spectra

Substrate cleaning, molecule deposition and low-temperature STM observations were carried out in ultra-high vacuum chambers, whose details are described elsewhere^54^,^55^. The sample temperature was ∼4.7 K for the STM/STS experiments described in this report. STS spectra were obtained using a lock-in amplifier with a modulation voltage of 1 mV superimposed onto the tunnelling bias voltage.

### Kondo mapping set-up

Kondo mapping was carried out by measuring the d*I/*d*V* curve in the vicinity of the Fermi level at the lattice points of a 64 × 64-grid. To obtain the data, the STM tip scans the surface with a movement along the *x-* and *y* directions similar to that for topography imaging. At each point of the grid, however, the system pauses, turns off the feedback loop and obtains the STS measurement in the vicinity of the Fermi level. Since it takes ∼10 s at each point, the total time for one mapping image is ca. 10 h. During data analysis, the amplitude of the Fano-shaped Kondo resonance is calculated at each point and a map of Δ*G/G*_0_ (the normalized conduction change, Δ*G* is the decrease in conduction at the Fano dip, while *G*_0_ is the conductance at Fermi level) is created.

### Theoretical calculations

First-principle calculations for determining the adsorption configuration and the STM simulation were performed using the VASP code with a plane wave basis set, and projector augmented wave (PAW) potentials to describe the behaviour of the valence electrons^56^,^57^. A generalized gradient Perdew–Burke–Ernzerh of exchange-correlation potential^58^ was used. The structures were relaxed until the forces were smaller than 0.05 eV Å^−1^. Due to the absence of dispersion forces in the local and semi-local exchange-correlation approximations, the molecule-surface distances of weak bonds such as van der Waals interactions remain controversial. Although this leads to ambiguity in the predicted charge transfer from the substrate to the molecule, the calculation results are accurate enough to enhance the understanding of the spin behaviour. The optimized structures were also used to calculate the spin-polarized partial density of states. The simulated STM images were formed using the Tersoff and Hamman theory^59^, and were visualized using a previously reported method^60^. DFT geometry optimizations were carried out for Cu-TPC, Cu-BCOD and Cu-Benzo at the Centre for High Performance Computing in Cape Town by using the B3LYP functional of the Gaussian 09 software package^61^ with 6–31G(d) basis sets. For VASP- and B3LYP-optimized structures, see Supplementary Data 3 and 4.

## Additional information

**Accession codes:** The X-ray crystallographic coordinates for structures reported in this Article have been deposited at the Cambridge Crystallographic Data Centre (CCDC), under deposition number CCDC 1400932. These data can be obtained free of charge from The Cambridge Crystallographic Data Centre via www.ccdc.cam.ac.uk/data_request/cif.

**How to cite this article:** Wu, F. *et al.* Modulation of the molecular spintronic properties of adsorbed copper corroles. *Nat. Commun.* 6:7547 doi: 10.1038/ncomms8547 (2015).

## Supplementary Material

Supplementary Information Supplementary Figures 1-11, Supplementary Notes 1-2, and Supplementary References

Supplementary Data 1 cif file for Cu-Benzo molecule.

Supplementary Data 2 The displacements of the twenty-three core ligand atoms.

Supplementary Data 3 Coordinates for the VASP Optimized Structures.

Supplementary Data 4 Coordinates for the B3LYP optimized structures.
