# Supplementary material for: Modulation of the molecular spintronic properties of adsorbed copper corroles
Source: Nat Commun. 2015 Jun 26;6:7547. doi: 10.1038/ncomms8547 (PMC4491828; doi:10.1038/ncomms8547)
Supplement: Supplementary Information — Supplementary Figures 1-11, Supplementary Notes 1-2, and Supplementary References [file ncomms8547-s1.pdf]

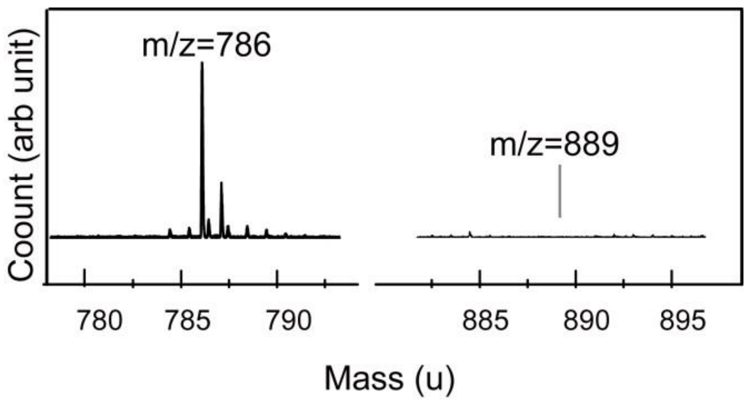

Supplementary Figure 1. ToF-SIMS data obtained for the Au(111) surface after the sublimation of the **Cu-BCOD** (mass 889 a.u.). During the sublimation process, **Cu-BCOD** was converted into **Cu-Benzo** (mass 786 a.u.).

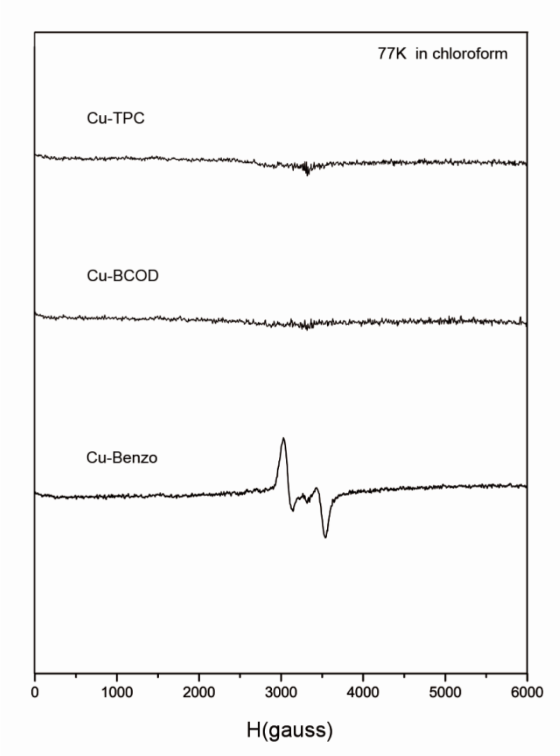

Supplementary Figure 2. The EPR spectrum obtained for **Cu-TPC, Cu-BCOD** and **Cu-Benzo** in frozen $CHCl_3$ at 77K.

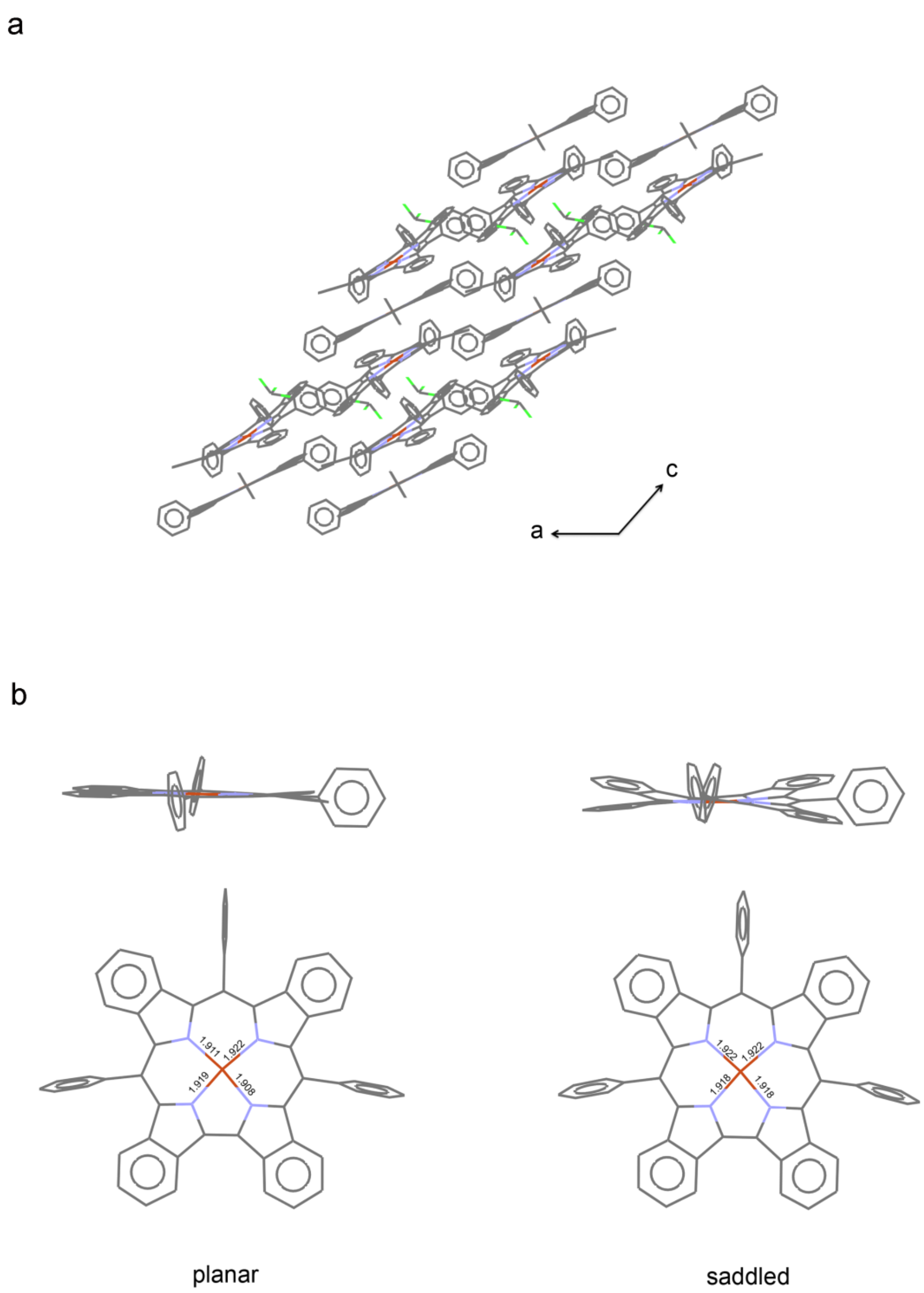

Supplementary Figure 3. (a) Crystal packing diagram of **Cu-benzo**, shown along with the crystallograhic b axis. **(b)** Side and top view with selected **Cu-N** bond lengths of planar and saddled **Cu-Benzo** in the crystal structure (H atoms omitted for clarity).

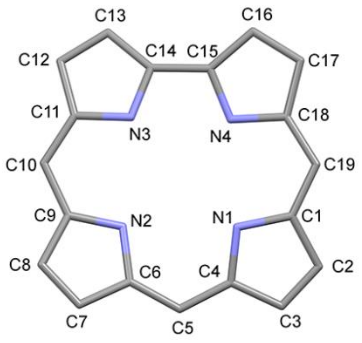

Supplementary Figure 4. The twenty three core ligand atoms of **Cu-TPC** and **Cu-Benzo** with labels.

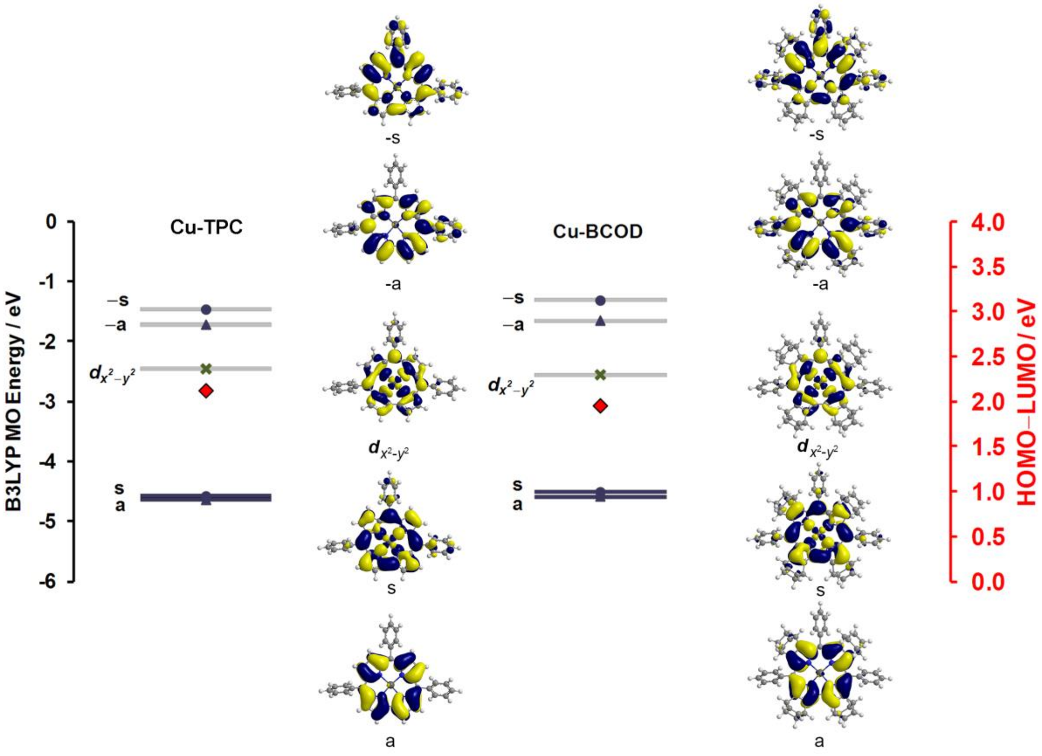

Supplementary Figure 5. The nodal patterns and energies of the frontier MOs of **Cu-TPC** and **Cu-BCOD** in DFT calculations carried out with the B3LYP functional and 6-31G(d) basis sets. Occupied and empty MOs are highlighted with blue and gray lines, respectively, and blue circles and triangles and green crosses are used to denote the **s** and **-s** MOs, **a** and **-a** MOs and the $d_{x^2-y^2}$ MOs, respectively. The predicted HOMO-LUMO gaps are denoted with red diamonds and are plotted against a secondary axis.

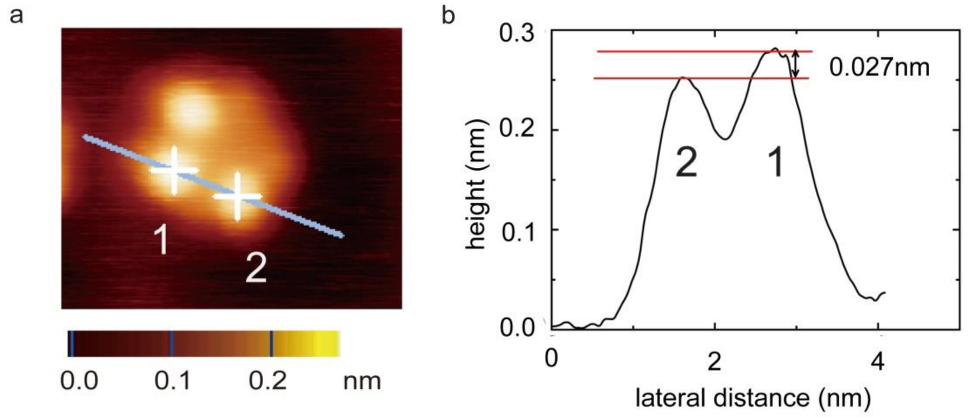

Supplementary Figure 6. **(a)** STM image of the **Cu-Benzo** monomer. The central *meso*-aryl ring is labelled with cross 1 and one of the side *meso*-aryl rings is labelled with cross 2. **(b)** The cross sectional height variation along the line connecting aryl rings 1 and 2.

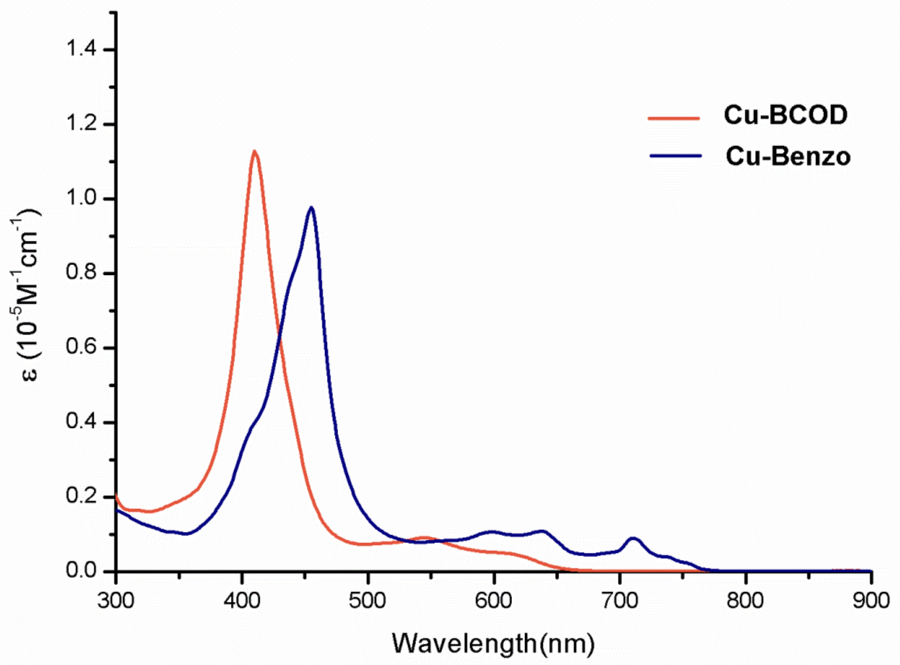

Supplementary Figure 7. UV-visible absorption spectra of **Cu-BCOD**(red)and **Cu-Benzo**(blue) in $CHCl_3$.

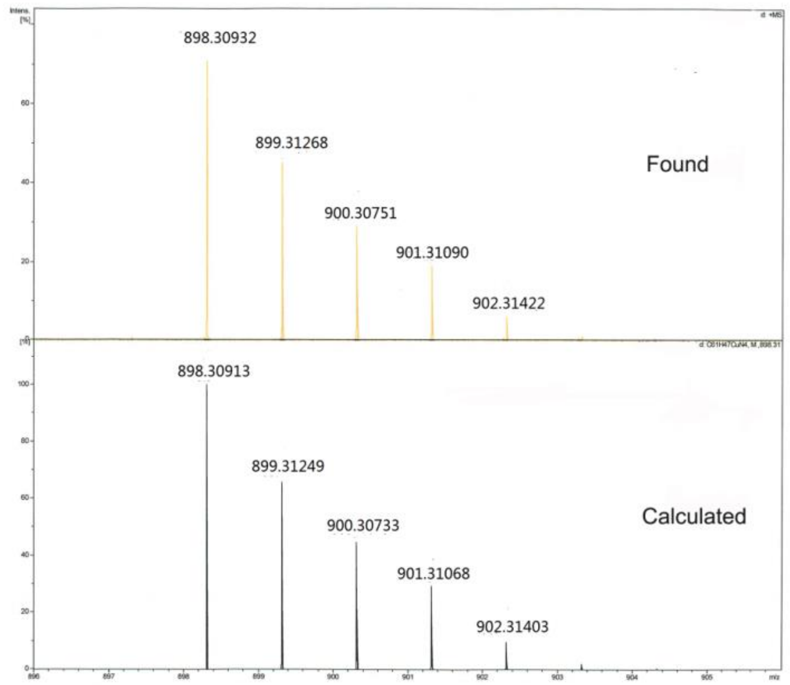

Supplementary Figure 8. HR-MS (MALDI-TOF) data for **Cu-BCOD**.

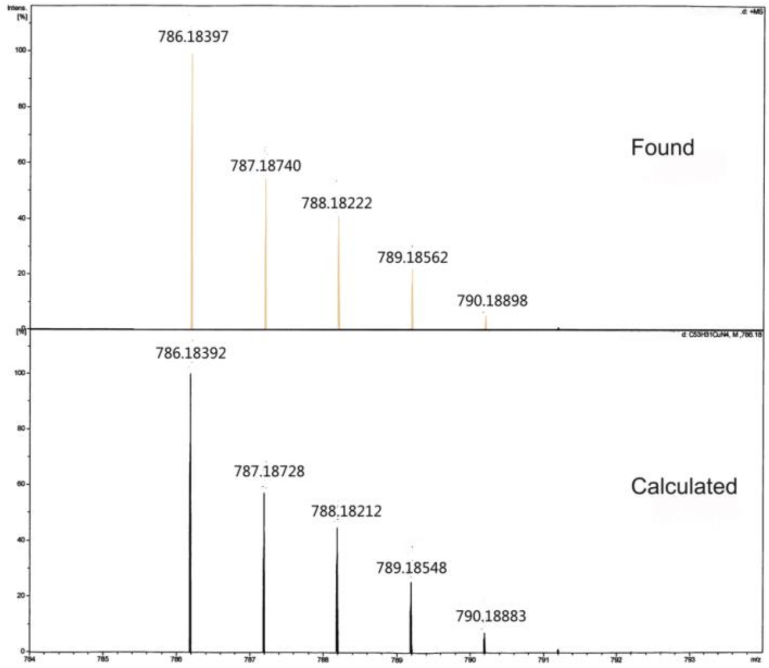

Supplementary Figure 9. HR-MS (MALDI-TOF) data for **Cu-Benzo**.

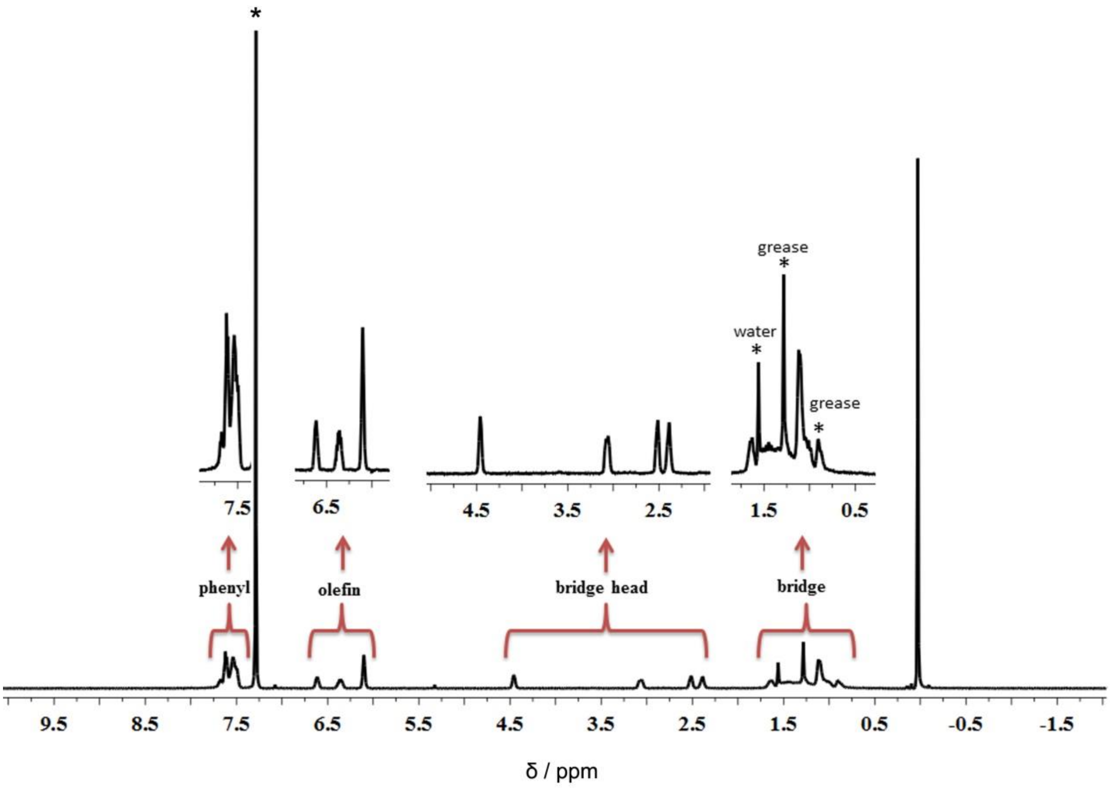

Supplementary Figure 10. ^{1}H NMR spectrum of **Cu-BCOD**. The solvent residual signal is marked with an asterisk.

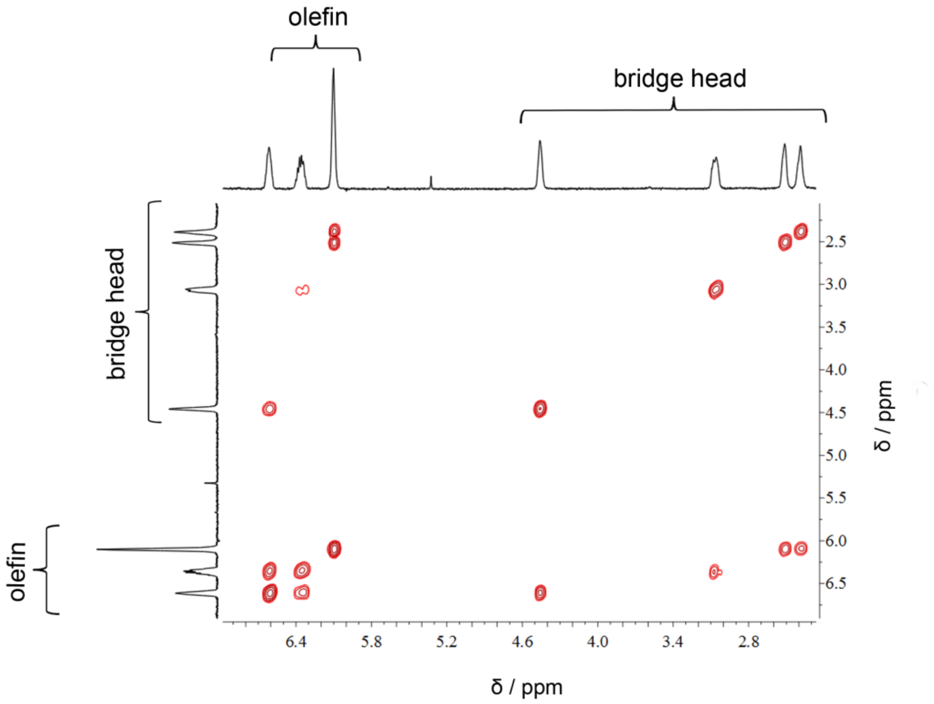

Supplementary Figure 11. $^1H^1H$-COSY NMR spectrum of **Cu-BCOD** in $CDCl_3$.

Supplementary note 1

The out-of-plane distortions of **Cu-TPC** and **Cu-Benzo**

An X-ray crystal structure obtained by slow diffusion of methanol into chloroform has been reported previously for **Cu-Benzo** prepared through a different synthetic route[1]. In this experiment, we obtained a similar crystal structure with two different conformations after slow diffusion of hexane into chloroform. The crystal data for **Cu-Benzo** we got, along with the **Cu-TPC** crystal structure reported by Brückner et al[2], are included to facilitate the explanation of the Au(111) surfaces. The enantiomeric inversion values of **Cu-TPC** and **Cu-Benzo** optimized structures are used in the clothes-line displays so a clear comparison can be made between the conformations of the structures.

Supplementary note 2

STM image of the tilted configuration of **Cu-Benzo** on Au(111)

In order to show the tilted configuration of **Cu-Benzo** on Au(111) that is predicted by theoretical calculation Kondo mapping can be carried out to provide a detailed topographic image. We measured the height variation between the central and side aryl rings. **Supplementary Figure 6** shows the cross sectional height variation along the line connecting aryl rings 1 and 2. There appears to be a ~0.3 Å difference between the heights of the two aryl rings. This pattern was observed in all of the monomers that we examined (~20 molecules). The height difference provides evidence for a tilted configuration with the position of the central aryl ring being the furthest from the surface.

Supplementary references

1. Pomarico, G., *et al.* Synthetic routes to 5, 10, 15-triaryl-tetrabenzocorroles. *J. Org. Chem.* **76**, 3765-3773 (2011).
2. Brückner, C., Briñas, R.P. & Krause Bauer, J.A. X-ray Structure and Variable Temperature NMR Spectra of [meso-Triarylcorrolato]copper(III). *Inorg. Chem.* **42**, 4495-4497 (2003).
