# Supplementary material for: Modulation of the molecular spintronic properties of adsorbed copper corroles
Source: Nat Commun. 2015 Jun 26;6:7547. doi: 10.1038/ncomms8547 (PMC4491828; doi:10.1038/ncomms8547)
Supplement: Supplementary Data 2 — The displacements of the twenty-three core ligand atoms. [file ncomms8547-s3.docx]

**The displacements of the twenty-three core ligand atoms**

The displacements of the twenty-three core ligand atoms from the core mean planes of the crystal structure and optimized structures calculated by using the B3LYP functional with 6-31G(d)  basis sets.

|  | Crystal structure displacements（Å） | | | Optimized structure displacements（Å） | | | |
| --- | --- | --- | --- | --- | --- | --- | --- |
|  | **CuTPC** | **Cu-Benzo** | C**u-Benzo** | **Cu-TPC** | **Cu-TPC** | **Cu-Benzo** | **Cu-Benzo** |
|  | (saddled) | (saddled) | (planar) | (singlet) | (triplet) | (singlet) | (triplet) |
| C14 | 0.030 | -0.001 | −0.014 | −0.036 | −0.076 | −0.068 | 0.026 |
| C13 | −0.113 | 0.313 | 0.002 | 0.177 | 0.065 | −0.470 | 0.114 |
| N3 | 0.107 | -0.069 | −0.012 | −0.215 | −0.130 | 0.140 | 0.000 |
| C12 | −0.224 | 0.451 | 0.044 | 0.203 | 0.116 | -0.552 | 0.119 |
| C11 | −0.112 | 0.171 | 0.018 | 0.001 | −0.006 | −0.189 | 0.045 |
| C10 | −0.195 | 0.118 | 0.044 | 0.075 | 0.060 | −0.169 | 0.015 |
| C9 | −0.091 | -0.064 | 0.025 | 0.052 | 0.046 | 0.045 | −0.025 |
| C8 | 0.243 | -0.272 | −0.038 | −0.195 | −0.057 | 0.519 | −0.096 |
| N2 | −0.202 | -0.031 | 0.005 | 0.208 | 0.084 | −0.157 | −0.003 |
| C7 | 0.360 | -0.339 | −0.083 | −0.222 | −0.079 | 0.593 | −0.109 |
| C6 | 0.080 | -0.152 | −0.024 | −0.009 | 0.015 | 0.152 | −0.039 |
| C5 | 0.116 | -0.014 | 0.000 | 0.005 | −0.001 | 0.000 | −0.000 |
| C4 | 0.007 | 0.105 | 0.024 | −0.003 | −0.004 | −0.152 | 0.039 |
| C3 | −0.274 | 0.369 | 0.083 | 0.185 | 0.026 | −0.593 | 0.108 |
| N1 | 0.160 | -0.038 | −0.005 | −0.166 | 0.011 | 0.157 | −0.003 |
| C2 | −0.292 | 0.358 | 0.038 | 0.170 | 0.029 | −0.519 | 0.096 |
| C1 | −0.019 | 0.095 | −0.025 | −0.010 | 0.032 | −0.045 | 0.025 |
| C19 | 0.012 | -0.039 | −0.044 | −0.030 | 0.032 | 0.169 | −0.015 |
| C18 | 0.011 | -0.171 | −0.018 | 0.003 | −0.041 | 0.189 | −0.045 |
| C17 | 0.267 | -0.305 | −0.044 | −0.150 | 0.069 | 0.552 | −0.119 |
| N4 | −0.197 | -0.105 | 0.012 | 0.151 | −0.146 | −0.140 | −0.000 |
| C16 | 0.294 | -0.262 | −0.002 | −0.172 | 0.035 | 0.470 | −0.115 |
| C15 | 0.034 | -0.119 | 0.014 | −0.024 | −0.088 | 0.069 | −0.026 |
