# Supplementary material for: Modulation of the molecular spintronic properties of adsorbed copper corroles
Source: Nat Commun. 2015 Jun 26;6:7547. doi: 10.1038/ncomms8547 (PMC4491828; doi:10.1038/ncomms8547)
Supplement: Supplementary Data 3 — Coordinates for the VASP Optimized Structures. [file ncomms8547-s4.docx]

**Coordinates for the VASP Optimized Structures**

Coordinates after optimization of **Cu-Benzo** and **Cu-TPC** molecules in vacuo. They are expressed using the periodic boundary conditions with the lattice vectors of (28.80, 0, 0) (0, 24.94, 0) (0, 0, 20.00).

| **Cu-Benzo** *in vacuo* | | | | **Cu-TPC** *in vacuo* | | | |
| --- | --- | --- | --- | --- | --- | --- | --- |
|  | *x* | *y* | *z* |  | *x* | *y* | *z* |
| C | 22.23 | 0.98 | 0.00 | C | 22.23 | 24.67 | 0.04 |
| C | 21.54 | 2.22 | 0.00 | C | 21.54 | 23.43 | 0.29 |
| C | 22.48 | 3.22 | 0.00 | C | 22.48 | 22.43 | 0.27 |
| C | 23.75 | 2.62 | 0.00 | C | 23.75 | 23.03 | 0.02 |
| C | 0.00 | 3.28 | 0.00 | C | 0.00 | 22.37 | 0.00 |
| C | 1.25 | 2.62 | 0.00 | C | 1.25 | 23.03 | 24.98 |
| C | 2.52 | 3.22 | 0.00 | C | 2.52 | 22.43 | 24.73 |
| C | 3.46 | 2.22 | 0.00 | C | 3.46 | 23.43 | 24.71 |
| C | 2.77 | 0.98 | 0.00 | C | 2.77 | 24.67 | 24.96 |
| C | 3.37 | 24.69 | 0.00 | C | 3.37 | 0.96 | 24.96 |
| C | 2.59 | 23.52 | 0.00 | C | 2.59 | 2.13 | 24.92 |
| C | 2.95 | 22.14 | 0.00 | C | 2.95 | 3.51 | 0.10 |
| C | 1.79 | 21.40 | 0.00 | C | 1.79 | 4.25 | 0.13 |
| C | 0.71 | 22.32 | 0.00 | C | 0.71 | 3.33 | 24.97 |
| C | 24.29 | 22.32 | 0.00 | C | 24.29 | 3.33 | 0.03 |
| C | 23.21 | 21.40 | 0.00 | C | 23.21 | 4.25 | 24.87 |
| C | 22.05 | 22.14 | 0.00 | C | 22.05 | 3.51 | 24.90 |
| C | 22.41 | 23.52 | 0.00 | C | 22.41 | 2.13 | 0.08 |
| C | 21.63 | 24.69 | 0.00 | C | 21.63 | 0.96 | 0.04 |
| C | 20.18 | 2.36 | 0.00 | C | 20.15 | 1.07 | 0.02 |
| C | 22.08 | 4.56 | 0.00 | C | 19.46 | 1.73 | 1.06 |
| C | 20.71 | 4.83 | 0.00 | C | 18.07 | 1.84 | 1.04 |
| C | 19.76 | 3.78 | 0.00 | C | 17.34 | 1.31 | 24.98 |
| C | 2.89 | 4.60 | 0.00 | C | 18.00 | 0.66 | 23.94 |
| C | 4.83 | 2.44 | 0.00 | C | 19.39 | 0.54 | 23.96 |
| C | 5.27 | 3.77 | 0.00 | C | 0.00 | 20.88 | 0.00 |
| C | 4.35 | 4.84 | 0.00 | C | 24.47 | 20.16 | 23.92 |
| C | 4.18 | 21.93 | 0.00 | C | 24.47 | 18.76 | 23.92 |
| C | 1.99 | 20.02 | 0.00 | C | 0.00 | 18.06 | 0.00 |
| C | 3.31 | 19.56 | 0.00 | C | 0.53 | 18.76 | 1.08 |
| C | 4.39 | 20.47 | 0.00 | C | 0.53 | 20.16 | 1.08 |
| C | 22.94 | 19.85 | 0.00 | C | 4.85 | 1.07 | 24.98 |
| C | 20.71 | 21.73 | 0.00 | C | 5.61 | 0.54 | 1.04 |
| C | 20.47 | 20.35 | 0.00 | C | 7.00 | 0.66 | 1.06 |
| C | 21.53 | 19.42 | 0.00 | C | 7.66 | 1.31 | 0.02 |
| C | 0.00 | 4.78 | 0.00 | C | 6.93 | 1.84 | 23.96 |
| C | 0.00 | 5.48 | 1.21 | C | 5.54 | 1.73 | 23.94 |
| C | 0.00 | 6.88 | 1.21 | N | 23.57 | 24.39 | 24.84 |
| C | 0.00 | 7.58 | 0.00 | N | 1.43 | 24.39 | 0.16 |
| C | 0.00 | 6.88 | 23.79 | N | 1.23 | 2.08 | 24.78 |
| C | 0.00 | 5.48 | 23.79 | N | 23.77 | 2.08 | 0.22 |
| C | 4.91 | 24.69 | 24.69 | H | 20.49 | 23.34 | 0.48 |
| C | 5.61 | 24.69 | 23.47 | H | 22.31 | 21.38 | 0.47 |
| C | 7.01 | 24.69 | 23.47 | H | 2.69 | 21.38 | 24.53 |
| C | 7.71 | 24.69 | 24.69 | H | 4.51 | 23.34 | 24.52 |
| C | 7.01 | 24.69 | 0.90 | H | 3.96 | 3.88 | 0.25 |
| C | 5.61 | 24.69 | 0.90 | H | 1.69 | 5.32 | 0.27 |
| C | 20.09 | 24.69 | 24.69 | H | 23.31 | 5.32 | 24.73 |
| C | 19.39 | 24.69 | 0.90 | H | 21.04 | 3.88 | 24.75 |
| C | 17.99 | 24.69 | 0.90 | H | 20.03 | 2.14 | 1.89 |
| C | 17.29 | 24.69 | 24.69 | H | 17.57 | 2.35 | 1.86 |
| C | 17.99 | 24.69 | 23.47 | H | 16.25 | 1.40 | 24.96 |
| C | 19.39 | 24.69 | 23.47 | H | 17.44 | 0.25 | 23.10 |
| N | 23.57 | 1.26 | 0.00 | H | 19.91 | 0.04 | 23.14 |
| N | 1.43 | 1.26 | 0.00 | H | 0.95 | 20.70 | 1.93 |
| N | 1.23 | 23.57 | 0.00 | H | 24.05 | 20.70 | 23.07 |
| N | 23.77 | 23.57 | 0.00 | H | 24.06 | 18.23 | 23.06 |
| H | 19.49 | 1.52 | 0.00 | H | 0.00 | 16.97 | 0.00 |
| H | 22.83 | 5.36 | 0.00 | H | 0.94 | 18.23 | 1.94 |
| H | 20.35 | 5.86 | 0.00 | H | 4.97 | 2.14 | 23.11 |
| H | 18.71 | 4.03 | 0.00 | H | 7.43 | 2.35 | 23.14 |
| H | 2.14 | 5.40 | 0.00 | H | 8.75 | 1.40 | 0.04 |
| H | 5.54 | 1.60 | 0.00 | H | 7.56 | 0.25 | 1.90 |
| H | 6.34 | 3.99 | 0.00 | H | 5.10 | 0.04 | 1.86 |
| H | 4.73 | 5.85 | 0.00 | Cu | 0.00 | 0.65 | 0.00 |
| H | 4.98 | 22.67 | 0.00 |  |  |  |  |
| H | 1.14 | 19.33 | 0.00 |  |  |  |  |
| H | 3.52 | 18.49 | 0.00 |  |  |  |  |
| H | 5.40 | 20.07 | 0.00 |  |  |  |  |
| H | 23.79 | 19.17 | 0.00 |  |  |  |  |
| H | 19.90 | 22.46 | 0.00 |  |  |  |  |
| H | 19.44 | 19.98 | 0.00 |  |  |  |  |
| H | 21.29 | 18.36 | 0.00 |  |  |  |  |
| H | 0.00 | 4.93 | 2.16 |  |  |  |  |
| H | 0.00 | 7.42 | 2.16 |  |  |  |  |
| H | 0.00 | 8.67 | 0.00 |  |  |  |  |
| H | 0.00 | 7.42 | 22.84 |  |  |  |  |
| H | 0.00 | 4.93 | 22.84 |  |  |  |  |
| H | 5.06 | 24.69 | 22.53 |  |  |  |  |
| H | 7.55 | 24.69 | 22.53 |  |  |  |  |
| H | 8.80 | 24.69 | 24.69 |  |  |  |  |
| H | 7.55 | 24.69 | 1.85 |  |  |  |  |
| H | 5.06 | 24.69 | 1.85 |  |  |  |  |
| H | 19.94 | 24.69 | 1.85 |  |  |  |  |
| H | 17.45 | 24.69 | 1.85 |  |  |  |  |
| H | 16.20 | 24.69 | 24.69 |  |  |  |  |
| H | 17.45 | 24.69 | 22.53 |  |  |  |  |
| H | 19.94 | 24.69 | 22.53 |  |  |  |  |
| Cu | 0.00 | 0.00 | 0.00 |  |  |  |  |
|  |  |  |  |  |  |  |  |

Coordinates after optimization for **Cu-Benzo** and **Cu-TPC** molecules placed on Au(111) (geometries are expressed using the periodic boundary conditions with the lattice vectors of (28.80, 0, 0) (0, 24.94, 0) (0, 0, 20.00)).

| **Cu-Benzo** on Au(111) | | | | **Cu-TPC** on Au(111) | | | |
| --- | --- | --- | --- | --- | --- | --- | --- |
|  | *x* | *y* | *z* |  | *x* | *y* | *z* |
| C | 25.97 | 1.03 | 13.58 | C | 26.02 | 24.64 | 12.51 |
| C | 25.22 | 2.31 | 13.59 | C | 25.33 | 23.38 | 12.73 |
| C | 26.19 | 3.36 | 13.62 | C | 26.26 | 22.36 | 12.71 |
| C | 27.52 | 2.70 | 13.63 | C | 27.57 | 22.98 | 12.48 |
| C | 28.79 | 3.36 | 13.67 | C | 0.03 | 22.30 | 12.50 |
| C | 1.26 | 2.70 | 13.63 | C | 1.30 | 22.98 | 12.41 |
| C | 2.59 | 3.36 | 13.62 | C | 2.59 | 22.36 | 12.19 |
| C | 3.57 | 2.31 | 13.59 | C | 3.54 | 23.39 | 12.18 |
| C | 2.82 | 1.03 | 13.58 | C | 2.84 | 24.64 | 12.39 |
| C | 3.38 | 24.66 | 13.58 | C | 3.46 | 1.01 | 12.48 |
| C | 2.60 | 23.47 | 13.58 | C | 2.66 | 2.20 | 12.39 |
| C | 3.03 | 22.06 | 13.59 | C | 3.01 | 3.61 | 12.51 |
| C | 1.83 | 21.24 | 13.61 | C | 1.82 | 4.36 | 12.42 |
| C | 0.70 | 22.19 | 13.60 | C | 0.75 | 3.41 | 12.28 |
| C | 28.08 | 22.19 | 13.61 | C | 28.11 | 3.41 | 12.32 |
| C | 26.95 | 21.24 | 13.62 | C | 27.02 | 4.35 | 12.23 |
| C | 25.75 | 22.06 | 13.60 | C | 25.83 | 3.60 | 12.32 |
| C | 26.18 | 23.47 | 13.59 | C | 26.20 | 2.19 | 12.47 |
| C | 25.40 | 24.67 | 13.58 | C | 25.41 | 1.00 | 12.54 |
| C | 23.83 | 2.62 | 13.58 | C | 23.92 | 1.10 | 12.66 |
| C | 25.77 | 4.72 | 13.64 | C | 23.29 | 2.07 | 13.49 |
| C | 24.39 | 5.02 | 13.64 | C | 21.88 | 2.21 | 13.52 |
| C | 23.43 | 3.98 | 13.61 | C | 21.06 | 1.37 | 12.73 |
| C | 3.01 | 4.72 | 13.65 | C | 21.66 | 0.33 | 12.00 |
| C | 4.96 | 2.62 | 13.58 | C | 23.07 | 0.21 | 11.94 |
| C | 5.36 | 3.97 | 13.61 | C | 0.03 | 20.81 | 12.64 |
| C | 4.40 | 5.01 | 13.64 | C | 27.93 | 20.01 | 11.87 |
| C | 4.32 | 21.46 | 13.59 | C | 27.93 | 18.60 | 11.97 |
| C | 1.95 | 19.83 | 13.63 | C | 0.05 | 17.94 | 12.80 |
| C | 3.24 | 19.25 | 13.64 | C | 0.91 | 18.71 | 13.62 |
| C | 4.41 | 20.05 | 13.62 | C | 0.89 | 20.13 | 13.55 |
| C | 26.83 | 19.83 | 13.64 | C | 4.94 | 1.12 | 12.64 |
| C | 24.46 | 21.46 | 13.60 | C | 5.67 | 0.21 | 13.47 |
| C | 24.36 | 20.06 | 13.62 | C | 7.09 | 0.25 | 13.53 |
| C | 25.54 | 19.25 | 13.64 | C | 7.82 | 1.20 | 12.78 |
| C | 28.79 | 4.87 | 13.71 | C | 7.10 | 2.19 | 12.06 |
| C | 28.80 | 5.59 | 14.94 | C | 5.69 | 2.14 | 11.99 |
| C | 28.80 | 7.01 | 14.94 | N | 27.39 | 24.36 | 12.34 |
| C | 0.00 | 7.72 | 13.71 | N | 1.47 | 24.37 | 12.52 |
| C | 28.80 | 7.01 | 12.50 | N | 1.28 | 2.13 | 12.20 |
| C | 28.80 | 5.60 | 12.50 | N | 27.59 | 2.13 | 12.48 |
| C | 4.89 | 24.55 | 13.56 | H | 24.26 | 23.26 | 12.90 |
| C | 5.58 | 24.55 | 12.32 | H | 26.07 | 21.30 | 12.85 |
| C | 6.98 | 24.57 | 12.27 | H | 2.76 | 21.30 | 12.02 |
| C | 7.75 | 24.60 | 13.46 | H | 4.61 | 23.28 | 12.00 |
| C | 7.08 | 24.56 | 14.70 | H | 4.01 | 4.02 | 12.66 |
| C | 5.66 | 24.54 | 14.76 | H | 1.72 | 5.45 | 12.47 |
| C | 23.89 | 24.56 | 13.56 | H | 27.11 | 5.43 | 12.10 |
| C | 23.12 | 24.54 | 14.76 | H | 24.81 | 3.97 | 12.23 |
| C | 21.70 | 24.56 | 14.70 | H | 23.91 | 2.69 | 14.15 |
| C | 21.03 | 24.59 | 13.46 | H | 21.43 | 2.97 | 14.17 |
| C | 21.80 | 24.57 | 12.27 | H | 19.97 | 1.51 | 12.70 |
| C | 23.20 | 24.55 | 12.32 | H | 21.04 | 24.56 | 11.43 |
| N | 27.34 | 1.32 | 13.59 | H | 23.52 | 24.34 | 11.35 |
| N | 1.44 | 1.32 | 13.59 | H | 1.51 | 20.71 | 14.23 |
| N | 1.23 | 23.49 | 13.59 | H | 27.20 | 20.51 | 11.22 |
| N | 27.55 | 23.49 | 13.59 | H | 27.19 | 18.03 | 11.38 |
| H | 23.06 | 1.85 | 13.55 | H | 0.09 | 16.84 | 12.80 |
| H | 26.49 | 5.55 | 13.66 | H | 1.57 | 18.21 | 14.33 |
| H | 24.07 | 6.07 | 13.66 | H | 5.17 | 2.88 | 11.37 |
| H | 22.36 | 4.22 | 13.61 | H | 7.65 | 2.99 | 11.54 |
| H | 2.30 | 5.54 | 13.66 | H | 8.91 | 1.19 | 12.77 |
| H | 5.72 | 1.85 | 13.55 | H | 7.62 | 24.48 | 14.17 |
| H | 6.43 | 4.21 | 13.61 | H | 5.12 | 24.43 | 14.09 |
| H | 4.72 | 6.06 | 13.66 | Cu | 0.03 | 0.69 | 12.35 |
| H | 5.22 | 22.08 | 13.57 | Au | 0.00 | 0.00 | 9.15 |
| H | 1.07 | 19.17 | 13.65 | Au | 2.88 | 0.00 | 9.15 |
| H | 3.35 | 18.15 | 13.65 | Au | 5.76 | 0.00 | 9.15 |
| H | 5.40 | 19.58 | 13.62 | Au | 8.64 | 0.00 | 9.15 |
| H | 27.71 | 19.17 | 13.65 | Au | 11.52 | 0.00 | 9.15 |
| H | 23.56 | 22.08 | 13.58 | Au | 14.40 | 0.00 | 9.15 |
| H | 23.37 | 19.59 | 13.62 | Au | 17.28 | 0.00 | 9.15 |
| H | 25.43 | 18.16 | 13.65 | Au | 20.16 | 0.00 | 9.15 |
| H | 28.80 | 5.04 | 15.89 | Au | 23.04 | 0.00 | 9.15 |
| H | 0.00 | 7.56 | 15.89 | Au | 25.92 | 0.00 | 9.15 |
| H | 0.00 | 8.82 | 13.70 | Au | 1.44 | 2.49 | 9.15 |
| H | 0.00 | 7.55 | 11.53 | Au | 4.32 | 2.49 | 9.15 |
| H | 28.79 | 5.04 | 11.54 | Au | 7.20 | 2.49 | 9.15 |
| H | 4.99 | 24.56 | 11.39 | Au | 10.08 | 2.49 | 9.15 |
| H | 7.49 | 24.59 | 11.29 | Au | 12.96 | 2.49 | 9.15 |
| H | 8.84 | 24.64 | 13.40 | Au | 15.84 | 2.49 | 9.15 |
| H | 7.66 | 24.57 | 15.63 | Au | 18.72 | 2.49 | 9.15 |
| H | 5.15 | 24.54 | 15.72 | Au | 21.60 | 2.49 | 9.15 |
| H | 23.63 | 24.54 | 15.72 | Au | 24.48 | 2.49 | 9.15 |
| H | 21.12 | 24.57 | 15.63 | Au | 27.36 | 2.49 | 9.15 |
| H | 19.94 | 24.64 | 13.40 | Au | 2.88 | 4.99 | 9.15 |
| H | 21.29 | 24.59 | 11.29 | Au | 5.76 | 4.99 | 9.15 |
| H | 23.79 | 24.56 | 11.39 | Au | 8.64 | 4.99 | 9.15 |
| Cu | 28.79 | 0.02 | 13.57 | Au | 11.52 | 4.99 | 9.15 |
| Au | 28.80 | 24.94 | 9.33 | Au | 14.40 | 4.99 | 9.15 |
| Au | 2.86 | 24.94 | 9.29 | Au | 17.28 | 4.99 | 9.15 |
| Au | 5.75 | 0.01 | 9.08 | Au | 20.16 | 4.99 | 9.15 |
| Au | 8.67 | 0.00 | 9.21 | Au | 23.04 | 4.99 | 9.15 |
| Au | 11.53 | 24.94 | 9.35 | Au | 25.92 | 4.99 | 9.15 |
| Au | 14.40 | 24.94 | 9.33 | Au | 0.00 | 4.99 | 9.15 |
| Au | 17.27 | 24.94 | 9.35 | Au | 4.32 | 7.48 | 9.15 |
| Au | 20.13 | 0.00 | 9.21 | Au | 7.20 | 7.48 | 9.15 |
| Au | 23.05 | 0.02 | 9.08 | Au | 10.08 | 7.48 | 9.15 |
| Au | 25.94 | 24.94 | 9.30 | Au | 12.96 | 7.48 | 9.15 |
| Au | 1.44 | 2.49 | 9.32 | Au | 15.84 | 7.48 | 9.15 |
| Au | 4.32 | 2.50 | 9.31 | Au | 18.72 | 7.48 | 9.15 |
| Au | 7.20 | 2.51 | 9.29 | Au | 21.60 | 7.48 | 9.15 |
| Au | 10.08 | 2.50 | 9.34 | Au | 24.48 | 7.48 | 9.15 |
| Au | 12.96 | 2.49 | 9.34 | Au | 27.36 | 7.48 | 9.15 |
| Au | 15.84 | 2.49 | 9.34 | Au | 1.44 | 7.48 | 9.15 |
| Au | 18.72 | 2.50 | 9.34 | Au | 5.76 | 9.98 | 9.15 |
| Au | 21.60 | 2.51 | 9.29 | Au | 8.64 | 9.98 | 9.15 |
| Au | 24.48 | 2.50 | 9.32 | Au | 11.52 | 9.98 | 9.15 |
| Au | 27.36 | 2.49 | 9.32 | Au | 14.40 | 9.98 | 9.15 |
| Au | 2.89 | 4.99 | 9.32 | Au | 17.28 | 9.98 | 9.15 |
| Au | 5.77 | 5.00 | 9.36 | Au | 20.16 | 9.98 | 9.15 |
| Au | 8.64 | 4.99 | 9.35 | Au | 23.04 | 9.98 | 9.15 |
| Au | 11.53 | 4.99 | 9.35 | Au | 25.92 | 9.98 | 9.15 |
| Au | 14.40 | 4.99 | 9.33 | Au | 0.00 | 9.98 | 9.15 |
| Au | 17.27 | 4.99 | 9.35 | Au | 2.88 | 9.98 | 9.15 |
| Au | 20.15 | 5.00 | 9.35 | Au | 7.20 | 12.47 | 9.15 |
| Au | 23.03 | 5.00 | 9.36 | Au | 10.08 | 12.47 | 9.15 |
| Au | 25.91 | 4.99 | 9.32 | Au | 12.96 | 12.47 | 9.15 |
| Au | 28.80 | 4.98 | 9.20 | Au | 15.84 | 12.47 | 9.15 |
| Au | 4.33 | 7.49 | 9.35 | Au | 18.72 | 12.47 | 9.15 |
| Au | 7.20 | 7.48 | 9.33 | Au | 21.60 | 12.47 | 9.15 |
| Au | 10.08 | 7.48 | 9.34 | Au | 24.48 | 12.47 | 9.15 |
| Au | 12.96 | 7.48 | 9.33 | Au | 27.36 | 12.47 | 9.15 |
| Au | 15.84 | 7.48 | 9.34 | Au | 1.44 | 12.47 | 9.15 |
| Au | 18.72 | 7.48 | 9.34 | Au | 4.32 | 12.47 | 9.15 |
| Au | 21.60 | 7.48 | 9.33 | Au | 8.64 | 14.96 | 9.15 |
| Au | 24.47 | 7.49 | 9.35 | Au | 11.52 | 14.96 | 9.15 |
| Au | 27.34 | 7.49 | 9.26 | Au | 14.40 | 14.96 | 9.15 |
| Au | 1.46 | 7.49 | 9.26 | Au | 17.28 | 14.96 | 9.15 |
| Au | 5.76 | 9.98 | 9.34 | Au | 20.16 | 14.96 | 9.15 |
| Au | 8.64 | 9.98 | 9.33 | Au | 23.04 | 14.96 | 9.15 |
| Au | 11.52 | 9.98 | 9.34 | Au | 25.92 | 14.96 | 9.15 |
| Au | 14.40 | 9.98 | 9.32 | Au | 0.00 | 14.96 | 9.15 |
| Au | 17.28 | 9.98 | 9.34 | Au | 2.88 | 14.96 | 9.15 |
| Au | 20.16 | 9.98 | 9.33 | Au | 5.76 | 14.96 | 9.15 |
| Au | 23.04 | 9.98 | 9.34 | Au | 10.08 | 17.46 | 9.15 |
| Au | 25.92 | 9.98 | 9.34 | Au | 12.96 | 17.46 | 9.15 |
| Au | 28.80 | 9.98 | 9.31 | Au | 15.84 | 17.46 | 9.15 |
| Au | 2.88 | 9.98 | 9.34 | Au | 18.72 | 17.46 | 9.15 |
| Au | 7.20 | 12.47 | 9.32 | Au | 21.60 | 17.46 | 9.15 |
| Au | 10.08 | 12.47 | 9.34 | Au | 24.48 | 17.46 | 9.15 |
| Au | 12.96 | 12.47 | 9.33 | Au | 27.36 | 17.46 | 9.15 |
| Au | 15.84 | 12.47 | 9.33 | Au | 1.44 | 17.46 | 9.15 |
| Au | 18.72 | 12.47 | 9.34 | Au | 4.32 | 17.46 | 9.15 |
| Au | 21.60 | 12.47 | 9.32 | Au | 7.20 | 17.46 | 9.15 |
| Au | 24.47 | 12.47 | 9.35 | Au | 11.52 | 19.95 | 9.15 |
| Au | 27.36 | 12.47 | 9.34 | Au | 14.40 | 19.95 | 9.15 |
| Au | 1.44 | 12.47 | 9.34 | Au | 17.28 | 19.95 | 9.15 |
| Au | 4.33 | 12.47 | 9.35 | Au | 20.16 | 19.95 | 9.15 |
| Au | 8.64 | 14.96 | 9.33 | Au | 23.04 | 19.95 | 9.15 |
| Au | 11.52 | 14.96 | 9.34 | Au | 25.92 | 19.95 | 9.15 |
| Au | 14.40 | 14.96 | 9.32 | Au | 0.00 | 19.95 | 9.15 |
| Au | 17.28 | 14.96 | 9.34 | Au | 2.88 | 19.95 | 9.15 |
| Au | 20.16 | 14.96 | 9.33 | Au | 5.76 | 19.95 | 9.15 |
| Au | 23.04 | 14.96 | 9.34 | Au | 8.64 | 19.95 | 9.15 |
| Au | 25.92 | 14.96 | 9.35 | Au | 12.96 | 22.45 | 9.15 |
| Au | 28.80 | 14.96 | 9.33 | Au | 15.84 | 22.45 | 9.15 |
| Au | 2.88 | 14.96 | 9.35 | Au | 18.72 | 22.45 | 9.15 |
| Au | 5.76 | 14.96 | 9.34 | Au | 21.60 | 22.45 | 9.15 |
| Au | 10.08 | 17.46 | 9.34 | Au | 24.48 | 22.45 | 9.15 |
| Au | 12.96 | 17.46 | 9.33 | Au | 27.36 | 22.45 | 9.15 |
| Au | 15.84 | 17.46 | 9.33 | Au | 1.44 | 22.45 | 9.15 |
| Au | 18.72 | 17.46 | 9.34 | Au | 4.32 | 22.45 | 9.15 |
| Au | 21.60 | 17.46 | 9.33 | Au | 7.20 | 22.45 | 9.15 |
| Au | 24.47 | 17.46 | 9.35 | Au | 10.08 | 22.45 | 9.15 |
| Au | 27.36 | 17.45 | 9.35 | Au | 0.00 | 23.28 | 6.80 |
| Au | 1.44 | 17.45 | 9.35 | Au | 2.88 | 23.28 | 6.80 |
| Au | 4.33 | 17.46 | 9.35 | Au | 5.76 | 23.28 | 6.80 |
| Au | 7.20 | 17.46 | 9.33 | Au | 8.64 | 23.28 | 6.80 |
| Au | 11.53 | 19.95 | 9.35 | Au | 11.52 | 23.28 | 6.80 |
| Au | 14.40 | 19.95 | 9.32 | Au | 14.40 | 23.28 | 6.80 |
| Au | 17.27 | 19.95 | 9.35 | Au | 17.28 | 23.28 | 6.80 |
| Au | 20.16 | 19.94 | 9.35 | Au | 20.16 | 23.28 | 6.80 |
| Au | 23.04 | 19.94 | 9.35 | Au | 23.04 | 23.28 | 6.80 |
| Au | 25.93 | 19.94 | 9.35 | Au | 25.92 | 23.28 | 6.80 |
| Au | 28.80 | 19.95 | 9.33 | Au | 1.44 | 0.83 | 6.80 |
| Au | 2.87 | 19.94 | 9.35 | Au | 4.32 | 0.83 | 6.80 |
| Au | 5.76 | 19.94 | 9.35 | Au | 7.20 | 0.83 | 6.80 |
| Au | 8.64 | 19.94 | 9.35 | Au | 10.08 | 0.83 | 6.80 |
| Au | 12.96 | 22.44 | 9.34 | Au | 12.96 | 0.83 | 6.80 |
| Au | 15.84 | 22.44 | 9.34 | Au | 15.84 | 0.83 | 6.80 |
| Au | 18.72 | 22.44 | 9.33 | Au | 18.72 | 0.83 | 6.80 |
| Au | 21.60 | 22.43 | 9.26 | Au | 21.60 | 0.83 | 6.80 |
| Au | 24.49 | 22.43 | 9.29 | Au | 24.48 | 0.83 | 6.80 |
| Au | 27.37 | 22.44 | 9.34 | Au | 27.36 | 0.83 | 6.80 |
| Au | 1.43 | 22.44 | 9.34 | Au | 2.88 | 3.33 | 6.80 |
| Au | 4.31 | 22.43 | 9.29 | Au | 5.76 | 3.33 | 6.80 |
| Au | 7.20 | 22.43 | 9.26 | Au | 8.64 | 3.33 | 6.80 |
| Au | 10.08 | 22.44 | 9.33 | Au | 11.52 | 3.33 | 6.80 |
| Au | 28.80 | 23.27 | 6.79 | Au | 14.40 | 3.33 | 6.80 |
| Au | 2.87 | 23.27 | 6.78 | Au | 17.28 | 3.33 | 6.80 |
| Au | 5.76 | 23.26 | 6.73 | Au | 20.16 | 3.33 | 6.80 |
| Au | 8.65 | 23.27 | 6.76 | Au | 23.04 | 3.33 | 6.80 |
| Au | 11.53 | 23.27 | 6.79 | Au | 25.92 | 3.33 | 6.80 |
| Au | 14.40 | 23.28 | 6.79 | Au | 0.00 | 3.33 | 6.80 |
| Au | 17.27 | 23.27 | 6.79 | Au | 4.32 | 5.82 | 6.80 |
| Au | 20.15 | 23.27 | 6.76 | Au | 7.20 | 5.82 | 6.80 |
| Au | 23.04 | 23.26 | 6.73 | Au | 10.08 | 5.82 | 6.80 |
| Au | 25.93 | 23.27 | 6.78 | Au | 12.96 | 5.82 | 6.80 |
| Au | 1.43 | 0.83 | 6.77 | Au | 15.84 | 5.82 | 6.80 |
| Au | 4.29 | 0.85 | 6.72 | Au | 18.72 | 5.82 | 6.80 |
| Au | 7.21 | 0.85 | 6.72 | Au | 21.60 | 5.82 | 6.80 |
| Au | 10.10 | 0.84 | 6.76 | Au | 24.48 | 5.82 | 6.80 |
| Au | 12.96 | 0.83 | 6.79 | Au | 27.36 | 5.82 | 6.80 |
| Au | 15.84 | 0.83 | 6.79 | Au | 1.44 | 5.82 | 6.80 |
| Au | 18.69 | 0.84 | 6.76 | Au | 5.76 | 8.31 | 6.80 |
| Au | 21.59 | 0.85 | 6.72 | Au | 8.64 | 8.31 | 6.80 |
| Au | 24.51 | 0.85 | 6.72 | Au | 11.52 | 8.31 | 6.80 |
| Au | 27.37 | 0.83 | 6.77 | Au | 14.40 | 8.31 | 6.80 |
| Au | 2.87 | 3.34 | 6.78 | Au | 17.28 | 8.31 | 6.80 |
| Au | 5.76 | 3.34 | 6.78 | Au | 20.16 | 8.31 | 6.80 |
| Au | 8.65 | 3.34 | 6.78 | Au | 23.04 | 8.31 | 6.80 |
| Au | 11.53 | 3.34 | 6.80 | Au | 25.92 | 8.31 | 6.80 |
| Au | 14.40 | 3.33 | 6.79 | Au | 0.00 | 8.31 | 6.80 |
| Au | 17.27 | 3.34 | 6.80 | Au | 2.88 | 8.31 | 6.80 |
| Au | 20.15 | 3.34 | 6.78 | Au | 7.20 | 10.81 | 6.80 |
| Au | 23.04 | 3.34 | 6.78 | Au | 10.08 | 10.81 | 6.80 |
| Au | 25.93 | 3.34 | 6.78 | Au | 12.96 | 10.81 | 6.80 |
| Au | 28.80 | 3.31 | 6.76 | Au | 15.84 | 10.81 | 6.80 |
| Au | 4.33 | 5.83 | 6.79 | Au | 18.72 | 10.81 | 6.80 |
| Au | 7.20 | 5.82 | 6.79 | Au | 21.60 | 10.81 | 6.80 |
| Au | 10.08 | 5.83 | 6.80 | Au | 24.48 | 10.81 | 6.80 |
| Au | 12.96 | 5.82 | 6.79 | Au | 27.36 | 10.81 | 6.80 |
| Au | 15.84 | 5.82 | 6.79 | Au | 1.44 | 10.81 | 6.80 |
| Au | 18.72 | 5.82 | 6.80 | Au | 4.32 | 10.81 | 6.80 |
| Au | 21.60 | 5.82 | 6.79 | Au | 8.64 | 13.30 | 6.80 |
| Au | 24.47 | 5.83 | 6.79 | Au | 11.52 | 13.30 | 6.80 |
| Au | 27.35 | 5.83 | 6.76 | Au | 14.40 | 13.30 | 6.80 |
| Au | 1.45 | 5.83 | 6.76 | Au | 17.28 | 13.30 | 6.80 |
| Au | 5.76 | 8.32 | 6.79 | Au | 20.16 | 13.30 | 6.80 |
| Au | 8.64 | 8.31 | 6.79 | Au | 23.04 | 13.30 | 6.80 |
| Au | 11.52 | 8.32 | 6.80 | Au | 25.92 | 13.30 | 6.80 |
| Au | 14.40 | 8.31 | 6.79 | Au | 0.00 | 13.30 | 6.80 |
| Au | 17.28 | 8.31 | 6.80 | Au | 2.88 | 13.30 | 6.80 |
| Au | 20.16 | 8.31 | 6.79 | Au | 5.76 | 13.30 | 6.80 |
| Au | 23.04 | 8.32 | 6.79 | Au | 10.08 | 15.80 | 6.80 |
| Au | 25.91 | 8.33 | 6.78 | Au | 12.96 | 15.80 | 6.80 |
| Au | 28.80 | 8.32 | 6.77 | Au | 15.84 | 15.80 | 6.80 |
| Au | 2.89 | 8.33 | 6.78 | Au | 18.72 | 15.80 | 6.80 |
| Au | 7.20 | 10.81 | 6.79 | Au | 21.60 | 15.80 | 6.80 |
| Au | 10.08 | 10.81 | 6.80 | Au | 24.48 | 15.80 | 6.80 |
| Au | 12.96 | 10.81 | 6.79 | Au | 27.36 | 15.80 | 6.80 |
| Au | 15.84 | 10.81 | 6.79 | Au | 1.44 | 15.80 | 6.80 |
| Au | 18.72 | 10.81 | 6.80 | Au | 4.32 | 15.80 | 6.80 |
| Au | 21.60 | 10.81 | 6.79 | Au | 7.20 | 15.80 | 6.80 |
| Au | 24.47 | 10.81 | 6.80 | Au | 11.52 | 18.29 | 6.80 |
| Au | 27.35 | 10.81 | 6.79 | Au | 14.40 | 18.29 | 6.80 |
| Au | 1.45 | 10.81 | 6.79 | Au | 17.28 | 18.29 | 6.80 |
| Au | 4.33 | 10.81 | 6.80 | Au | 20.16 | 18.29 | 6.80 |
| Au | 8.64 | 13.30 | 6.79 | Au | 23.04 | 18.29 | 6.80 |
| Au | 11.52 | 13.30 | 6.80 | Au | 25.92 | 18.29 | 6.80 |
| Au | 14.40 | 13.30 | 6.79 | Au | 0.00 | 18.29 | 6.80 |
| Au | 17.28 | 13.30 | 6.80 | Au | 2.88 | 18.29 | 6.80 |
| Au | 20.16 | 13.30 | 6.79 | Au | 5.76 | 18.29 | 6.80 |
| Au | 23.04 | 13.30 | 6.79 | Au | 8.64 | 18.29 | 6.80 |
| Au | 25.92 | 13.30 | 6.80 | Au | 12.96 | 20.78 | 6.80 |
| Au | 28.80 | 13.30 | 6.79 | Au | 15.84 | 20.78 | 6.80 |
| Au | 2.88 | 13.30 | 6.80 | Au | 18.72 | 20.78 | 6.80 |
| Au | 5.76 | 13.30 | 6.79 | Au | 21.60 | 20.78 | 6.80 |
| Au | 10.08 | 15.79 | 6.80 | Au | 24.48 | 20.78 | 6.80 |
| Au | 12.96 | 15.79 | 6.79 | Au | 27.36 | 20.78 | 6.80 |
| Au | 15.84 | 15.79 | 6.79 | Au | 1.44 | 20.78 | 6.80 |
| Au | 18.72 | 15.79 | 6.80 | Au | 4.32 | 20.78 | 6.80 |
| Au | 21.60 | 15.80 | 6.79 | Au | 7.20 | 20.78 | 6.80 |
| Au | 24.48 | 15.79 | 6.80 | Au | 10.08 | 20.78 | 6.80 |
| Au | 27.36 | 15.79 | 6.79 | Au | 0.00 | 21.62 | 4.45 |
| Au | 1.44 | 15.79 | 6.79 | Au | 2.88 | 21.62 | 4.45 |
| Au | 4.32 | 15.79 | 6.80 | Au | 5.76 | 21.62 | 4.45 |
| Au | 7.20 | 15.79 | 6.79 | Au | 8.64 | 21.62 | 4.45 |
| Au | 11.52 | 18.29 | 6.80 | Au | 11.52 | 21.62 | 4.45 |
| Au | 14.40 | 18.29 | 6.79 | Au | 14.40 | 21.62 | 4.45 |
| Au | 17.28 | 18.29 | 6.80 | Au | 17.28 | 21.62 | 4.45 |
| Au | 20.16 | 18.29 | 6.79 | Au | 20.16 | 21.62 | 4.45 |
| Au | 23.04 | 18.28 | 6.79 | Au | 23.04 | 21.62 | 4.45 |
| Au | 25.92 | 18.28 | 6.80 | Au | 25.92 | 21.62 | 4.45 |
| Au | 28.80 | 18.29 | 6.79 | Au | 1.44 | 24.11 | 4.45 |
| Au | 2.88 | 18.28 | 6.80 | Au | 4.32 | 24.11 | 4.45 |
| Au | 5.76 | 18.28 | 6.79 | Au | 7.20 | 24.11 | 4.45 |
| Au | 8.64 | 18.29 | 6.79 | Au | 10.08 | 24.11 | 4.45 |
| Au | 12.96 | 20.78 | 6.79 | Au | 12.96 | 24.11 | 4.45 |
| Au | 15.84 | 20.78 | 6.79 | Au | 15.84 | 24.11 | 4.45 |
| Au | 18.72 | 20.78 | 6.79 | Au | 18.72 | 24.11 | 4.45 |
| Au | 21.60 | 20.77 | 6.77 | Au | 21.60 | 24.11 | 4.45 |
| Au | 24.48 | 20.77 | 6.78 | Au | 24.48 | 24.11 | 4.45 |
| Au | 27.36 | 20.78 | 6.79 | Au | 27.36 | 24.11 | 4.45 |
| Au | 1.44 | 20.78 | 6.79 | Au | 2.88 | 1.66 | 4.45 |
| Au | 4.32 | 20.76 | 6.78 | Au | 5.76 | 1.66 | 4.45 |
| Au | 7.20 | 20.77 | 6.77 | Au | 8.64 | 1.66 | 4.45 |
| Au | 10.08 | 20.78 | 6.79 | Au | 11.52 | 1.66 | 4.45 |
| Au | 28.80 | 21.61 | 4.24 | Au | 14.40 | 1.66 | 4.45 |
| Au | 2.88 | 21.61 | 4.25 | Au | 17.28 | 1.66 | 4.45 |
| Au | 5.76 | 21.61 | 4.24 | Au | 20.16 | 1.66 | 4.45 |
| Au | 8.64 | 21.61 | 4.25 | Au | 23.04 | 1.66 | 4.45 |
| Au | 11.52 | 21.61 | 4.25 | Au | 25.92 | 1.66 | 4.45 |
| Au | 14.40 | 21.61 | 4.25 | Au | 0.00 | 1.66 | 4.45 |
| Au | 17.28 | 21.61 | 4.25 | Au | 4.32 | 4.16 | 4.45 |
| Au | 20.16 | 21.61 | 4.25 | Au | 7.20 | 4.16 | 4.45 |
| Au | 23.04 | 21.61 | 4.24 | Au | 10.08 | 4.16 | 4.45 |
| Au | 25.92 | 21.61 | 4.24 | Au | 12.96 | 4.16 | 4.45 |
| Au | 1.44 | 24.11 | 4.24 | Au | 15.84 | 4.16 | 4.45 |
| Au | 4.32 | 24.11 | 4.23 | Au | 18.72 | 4.16 | 4.45 |
| Au | 7.20 | 24.11 | 4.23 | Au | 21.60 | 4.16 | 4.45 |
| Au | 10.08 | 24.11 | 4.24 | Au | 24.48 | 4.16 | 4.45 |
| Au | 12.96 | 24.11 | 4.25 | Au | 27.36 | 4.16 | 4.45 |
| Au | 15.84 | 24.11 | 4.25 | Au | 1.44 | 4.16 | 4.45 |
| Au | 18.72 | 24.11 | 4.24 | Au | 5.76 | 6.65 | 4.45 |
| Au | 21.60 | 24.11 | 4.23 | Au | 8.64 | 6.65 | 4.45 |
| Au | 24.48 | 24.11 | 4.23 | Au | 11.52 | 6.65 | 4.45 |
| Au | 27.36 | 24.11 | 4.24 | Au | 14.40 | 6.65 | 4.45 |
| Au | 2.88 | 1.67 | 4.23 | Au | 17.28 | 6.65 | 4.45 |
| Au | 5.76 | 1.67 | 4.23 | Au | 20.16 | 6.65 | 4.45 |
| Au | 8.64 | 1.67 | 4.24 | Au | 23.04 | 6.65 | 4.45 |
| Au | 11.52 | 1.67 | 4.24 | Au | 25.92 | 6.65 | 4.45 |
| Au | 14.40 | 1.66 | 4.25 | Au | 0.00 | 6.65 | 4.45 |
| Au | 17.28 | 1.67 | 4.24 | Au | 2.88 | 6.65 | 4.45 |
| Au | 20.16 | 1.67 | 4.23 | Au | 7.20 | 9.15 | 4.45 |
| Au | 23.04 | 1.67 | 4.23 | Au | 10.08 | 9.15 | 4.45 |
| Au | 25.92 | 1.67 | 4.23 | Au | 12.96 | 9.15 | 4.45 |
| Au | 28.80 | 1.66 | 4.23 | Au | 15.84 | 9.15 | 4.45 |
| Au | 4.32 | 4.16 | 4.24 | Au | 18.72 | 9.15 | 4.45 |
| Au | 7.20 | 4.16 | 4.25 | Au | 21.60 | 9.15 | 4.45 |
| Au | 10.08 | 4.16 | 4.25 | Au | 24.48 | 9.15 | 4.45 |
| Au | 12.96 | 4.16 | 4.25 | Au | 27.36 | 9.15 | 4.45 |
| Au | 15.84 | 4.16 | 4.25 | Au | 1.44 | 9.15 | 4.45 |
| Au | 18.72 | 4.16 | 4.25 | Au | 4.32 | 9.15 | 4.45 |
| Au | 21.60 | 4.16 | 4.25 | Au | 8.64 | 11.64 | 4.45 |
| Au | 24.48 | 4.16 | 4.24 | Au | 11.52 | 11.64 | 4.45 |
| Au | 27.36 | 4.16 | 4.24 | Au | 14.40 | 11.64 | 4.45 |
| Au | 1.44 | 4.16 | 4.24 | Au | 17.28 | 11.64 | 4.45 |
| Au | 5.76 | 6.65 | 4.24 | Au | 20.16 | 11.64 | 4.45 |
| Au | 8.64 | 6.65 | 4.25 | Au | 23.04 | 11.64 | 4.45 |
| Au | 11.52 | 6.65 | 4.25 | Au | 25.92 | 11.64 | 4.45 |
| Au | 14.40 | 6.65 | 4.25 | Au | 0.00 | 11.64 | 4.45 |
| Au | 17.28 | 6.65 | 4.25 | Au | 2.88 | 11.64 | 4.45 |
| Au | 20.16 | 6.65 | 4.25 | Au | 5.76 | 11.64 | 4.45 |
| Au | 23.04 | 6.65 | 4.24 | Au | 10.08 | 14.13 | 4.45 |
| Au | 25.92 | 6.65 | 4.24 | Au | 12.96 | 14.13 | 4.45 |
| Au | 28.80 | 6.65 | 4.24 | Au | 15.84 | 14.13 | 4.45 |
| Au | 2.88 | 6.65 | 4.24 | Au | 18.72 | 14.13 | 4.45 |
| Au | 7.20 | 9.15 | 4.25 | Au | 21.60 | 14.13 | 4.45 |
| Au | 10.08 | 9.15 | 4.25 | Au | 24.48 | 14.13 | 4.45 |
| Au | 12.96 | 9.15 | 4.25 | Au | 27.36 | 14.13 | 4.45 |
| Au | 15.84 | 9.14 | 4.25 | Au | 1.44 | 14.13 | 4.45 |
| Au | 18.72 | 9.15 | 4.25 | Au | 4.32 | 14.13 | 4.45 |
| Au | 21.60 | 9.15 | 4.25 | Au | 7.20 | 14.13 | 4.45 |
| Au | 24.48 | 9.15 | 4.24 | Au | 11.52 | 16.63 | 4.45 |
| Au | 27.36 | 9.15 | 4.24 | Au | 14.40 | 16.63 | 4.45 |
| Au | 1.44 | 9.15 | 4.24 | Au | 17.28 | 16.63 | 4.45 |
| Au | 4.32 | 9.15 | 4.24 | Au | 20.16 | 16.63 | 4.45 |
| Au | 8.64 | 11.64 | 4.25 | Au | 23.04 | 16.63 | 4.45 |
| Au | 11.52 | 11.64 | 4.25 | Au | 25.92 | 16.63 | 4.45 |
| Au | 14.40 | 11.64 | 4.25 | Au | 0.00 | 16.63 | 4.45 |
| Au | 17.28 | 11.64 | 4.25 | Au | 2.88 | 16.63 | 4.45 |
| Au | 20.16 | 11.64 | 4.25 | Au | 5.76 | 16.63 | 4.45 |
| Au | 23.04 | 11.64 | 4.25 | Au | 8.64 | 16.63 | 4.45 |
| Au | 25.92 | 11.64 | 4.25 | Au | 12.96 | 19.12 | 4.45 |
| Au | 28.80 | 11.64 | 4.25 | Au | 15.84 | 19.12 | 4.45 |
| Au | 2.88 | 11.64 | 4.25 | Au | 18.72 | 19.12 | 4.45 |
| Au | 5.76 | 11.64 | 4.25 | Au | 21.60 | 19.12 | 4.45 |
| Au | 10.08 | 14.13 | 4.25 | Au | 24.48 | 19.12 | 4.45 |
| Au | 12.96 | 14.13 | 4.25 | Au | 27.36 | 19.12 | 4.45 |
| Au | 15.84 | 14.13 | 4.25 | Au | 1.44 | 19.12 | 4.45 |
| Au | 18.72 | 14.13 | 4.25 | Au | 4.32 | 19.12 | 4.45 |
| Au | 21.60 | 14.13 | 4.25 | Au | 7.20 | 19.12 | 4.45 |
| Au | 24.48 | 14.13 | 4.25 | Au | 10.08 | 19.12 | 4.45 |
| Au | 27.36 | 14.13 | 4.25 |  |  |  |  |
| Au | 1.44 | 14.13 | 4.25 |  |  |  |  |
| Au | 4.32 | 14.13 | 4.25 |  |  |  |  |
| Au | 7.20 | 14.13 | 4.25 |  |  |  |  |
| Au | 11.52 | 16.63 | 4.25 |  |  |  |  |
| Au | 14.40 | 16.63 | 4.25 |  |  |  |  |
| Au | 17.28 | 16.63 | 4.25 |  |  |  |  |
| Au | 20.16 | 16.62 | 4.25 |  |  |  |  |
| Au | 23.04 | 16.62 | 4.25 |  |  |  |  |
| Au | 25.92 | 16.63 | 4.25 |  |  |  |  |
| Au | 28.80 | 16.63 | 4.25 |  |  |  |  |
| Au | 2.88 | 16.63 | 4.25 |  |  |  |  |
| Au | 5.76 | 16.62 | 4.25 |  |  |  |  |
| Au | 8.64 | 16.63 | 4.25 |  |  |  |  |
| Au | 12.96 | 19.12 | 4.25 |  |  |  |  |
| Au | 15.84 | 19.12 | 4.25 |  |  |  |  |
| Au | 18.72 | 19.12 | 4.25 |  |  |  |  |
| Au | 21.60 | 19.12 | 4.24 |  |  |  |  |
| Au | 24.48 | 19.12 | 4.24 |  |  |  |  |
| Au | 27.36 | 19.12 | 4.25 |  |  |  |  |
| Au | 1.44 | 19.12 | 4.25 |  |  |  |  |
| Au | 4.32 | 19.12 | 4.24 |  |  |  |  |
| Au | 7.20 | 19.12 | 4.24 |  |  |  |  |
| Au | 10.08 | 19.12 | 4.25 |  |  |  |  |

The coordinates of the **Cu-Benzo** trimer (left), and the **Cu-TPC** chains on Au(111) (right) *in vacuo* (geometries are expressed using the periodic boundary conditions with the lattice vectors of (40.32, 0, 0) (0, 34.92, 0) (0, 0, 20.00) for the former, and (28.80, 0, 0) (0, 24.94, 0) (0, 0, 20.00) for the latter).

| **Cu-Benzo** trimer *in vacuo* | | | | **Cu-TPC** chains on Au(111) | | | |
| --- | --- | --- | --- | --- | --- | --- | --- |
|  | *x* | *y* | *z* |  | *x* | *y* | *z* |
| C | 6.85 | 12.75 | 19.92 | C | 2.97 | 0.39 | 2.08 |
| C | 6.85 | 11.28 | 19.94 | C | 3.94 | 1.47 | 2.15 |
| C | 5.49 | 10.84 | 19.98 | C | 3.24 | 2.66 | 2.03 |
| C | 4.67 | 12.08 | 19.98 | C | 1.83 | 2.32 | 1.98 |
| C | 3.25 | 12.16 | 0.00 | C | 0.74 | 3.23 | 2.02 |
| C | 2.46 | 13.34 | 19.99 | C | 28.17 | 2.85 | 2.03 |
| C | 0.99 | 13.41 | 19.98 | C | 27.03 | 3.76 | 1.97 |
| C | 0.65 | 14.81 | 19.94 | C | 25.89 | 2.98 | 1.86 |
| C | 1.93 | 15.55 | 19.93 | C | 26.30 | 1.59 | 1.93 |
| C | 2.10 | 16.97 | 19.89 | C | 25.43 | 0.46 | 1.93 |
| C | 3.37 | 17.61 | 19.92 | C | 25.94 | 24.07 | 1.91 |
| C | 3.71 | 19.04 | 19.95 | C | 25.27 | 22.81 | 2.10 |
| C | 5.15 | 19.15 | 19.98 | C | 26.25 | 21.81 | 2.15 |
| C | 5.66 | 17.77 | 19.99 | C | 27.53 | 22.46 | 1.98 |
| C | 6.90 | 17.07 | 19.99 | C | 0.15 | 22.16 | 2.02 |
| C | 8.35 | 17.32 | 19.99 | C | 1.07 | 21.05 | 1.84 |
| C | 8.99 | 16.02 | 19.95 | C | 2.36 | 21.56 | 1.90 |
| C | 7.92 | 15.01 | 19.94 | C | 2.25 | 22.98 | 2.11 |
| C | 8.00 | 13.59 | 19.89 | C | 3.28 | 23.94 | 2.11 |
| C | 7.91 | 10.35 | 19.95 | C | 4.70 | 23.47 | 2.09 |
| C | 5.22 | 9.44 | 0.01 | C | 5.19 | 22.62 | 3.11 |
| C | 6.29 | 8.52 | 0.01 | C | 6.54 | 22.18 | 3.08 |
| C | 7.63 | 8.97 | 19.99 | C | 7.38 | 22.55 | 2.01 |
| C | 40.28 | 12.43 | 0.01 | C | 6.88 | 23.37 | 0.97 |
| C | 39.60 | 15.21 | 19.94 | C | 5.55 | 23.83 | 1.01 |
| C | 38.60 | 14.22 | 19.98 | C | 1.08 | 4.69 | 2.04 |
| C | 38.93 | 12.84 | 0.01 | C | 1.80 | 5.25 | 3.13 |
| C | 2.89 | 20.20 | 19.96 | C | 2.15 | 6.61 | 3.12 |
| C | 5.75 | 20.44 | 0.01 | C | 1.82 | 7.42 | 2.01 |
| C | 4.93 | 21.58 | 0.01 | C | 1.11 | 6.87 | 0.92 |
| C | 3.51 | 21.46 | 19.99 | C | 0.73 | 5.51 | 0.94 |
| C | 9.16 | 18.49 | 0.01 | C | 23.95 | 0.68 | 1.99 |
| C | 10.41 | 15.91 | 19.94 | C | 23.35 | 1.41 | 3.06 |
| C | 11.19 | 17.08 | 19.98 | C | 21.95 | 1.60 | 3.09 |
| C | 10.57 | 18.36 | 0.01 | C | 21.13 | 1.10 | 2.05 |
| C | 2.58 | 10.82 | 0.01 | C | 21.72 | 0.37 | 0.99 |
| C | 2.36 | 10.15 | 1.24 | C | 23.11 | 0.15 | 0.97 |
| C | 2.01 | 8.79 | 1.23 | C | 25.83 | 14.60 | 2.05 |
| C | 1.88 | 8.12 | 20.00 | C | 24.87 | 13.53 | 2.28 |
| C | 2.02 | 8.79 | 18.77 | C | 25.58 | 12.34 | 2.27 |
| C | 2.36 | 10.16 | 18.77 | C | 26.98 | 12.68 | 2.04 |
| C | 0.84 | 17.81 | 19.81 | C | 28.06 | 11.77 | 2.01 |
| C | 0.26 | 18.13 | 18.56 | C | 0.64 | 12.14 | 1.97 |
| C | 39.34 | 18.81 | 18.50 | C | 1.76 | 11.26 | 1.72 |
| C | 38.68 | 19.18 | 19.70 | C | 2.91 | 12.03 | 1.72 |
| C | 39.27 | 18.88 | 0.95 | C | 2.50 | 13.40 | 1.97 |
| C | 0.19 | 18.20 | 1.01 | C | 3.37 | 14.54 | 1.97 |
| C | 9.40 | 13.02 | 19.81 | C | 2.86 | 15.86 | 1.93 |
| C | 10.14 | 12.80 | 1.01 | C | 3.53 | 17.13 | 2.10 |
| C | 11.51 | 12.45 | 0.95 | C | 2.55 | 18.13 | 2.14 |
| C | 12.15 | 12.29 | 19.70 | C | 1.27 | 17.47 | 1.98 |
| C | 11.41 | 12.45 | 18.51 | C | 28.65 | 17.78 | 2.02 |
| C | 10.04 | 12.81 | 18.56 | C | 27.73 | 18.88 | 1.84 |
| C | 37.43 | 1.00 | 19.92 | C | 26.44 | 18.37 | 1.89 |
| C | 36.67 | 2.27 | 19.94 | C | 26.55 | 16.96 | 2.10 |
| C | 37.63 | 3.33 | 19.98 | C | 25.52 | 15.99 | 2.07 |
| C | 38.97 | 2.69 | 19.98 | C | 24.10 | 16.46 | 2.07 |
| C | 40.23 | 3.34 | 0.01 | C | 23.61 | 17.31 | 3.10 |
| C | 1.18 | 2.70 | 20.00 | C | 22.27 | 17.76 | 3.08 |
| C | 2.49 | 3.36 | 19.99 | C | 21.42 | 17.39 | 2.01 |
| C | 3.49 | 2.33 | 19.95 | C | 21.91 | 16.57 | 0.97 |
| C | 2.75 | 1.04 | 19.93 | C | 23.24 | 16.10 | 1.00 |
| C | 3.32 | 34.66 | 19.89 | C | 27.72 | 10.31 | 2.04 |
| C | 2.56 | 33.46 | 19.93 | C | 26.99 | 9.75 | 0.96 |
| C | 3.01 | 32.06 | 19.95 | C | 26.64 | 8.39 | 0.97 |
| C | 1.82 | 31.23 | 19.98 | C | 26.98 | 7.59 | 2.08 |
| C | 0.68 | 32.15 | 19.99 | C | 27.69 | 8.14 | 3.17 |
| C | 39.57 | 32.15 | 19.99 | C | 28.07 | 9.50 | 3.14 |
| C | 38.45 | 31.20 | 19.98 | C | 4.85 | 14.32 | 2.01 |
| C | 37.24 | 32.01 | 19.95 | C | 5.46 | 13.59 | 3.07 |
| C | 37.67 | 33.42 | 19.93 | C | 6.86 | 13.39 | 3.09 |
| C | 36.88 | 34.61 | 19.89 | C | 7.67 | 13.90 | 2.05 |
| C | 35.27 | 2.57 | 19.94 | C | 7.08 | 14.63 | 1.00 |
| C | 37.19 | 4.68 | 0.01 | C | 5.68 | 14.85 | 0.98 |
| C | 35.81 | 4.97 | 0.01 | N | 1.69 | 0.94 | 1.95 |
| C | 34.86 | 3.92 | 19.98 | N | 27.70 | 1.54 | 2.05 |
| C | 2.87 | 4.72 | 0.02 | N | 27.30 | 23.82 | 1.80 |
| C | 4.86 | 2.70 | 19.93 | N | 0.91 | 23.30 | 2.22 |
| C | 5.21 | 4.07 | 19.97 | N | 27.11 | 14.06 | 1.89 |
| C | 4.22 | 5.08 | 0.01 | N | 1.11 | 13.45 | 2.13 |
| C | 4.31 | 31.48 | 19.96 | N | 1.50 | 16.12 | 1.80 |
| C | 1.97 | 29.81 | 0.01 | N | 27.89 | 16.63 | 2.21 |
| C | 3.27 | 29.25 | 0.01 | H | 5.02 | 1.32 | 2.28 |
| C | 4.43 | 30.08 | 19.99 | H | 3.65 | 3.67 | 1.98 |
| C | 38.32 | 29.78 | 0.01 | H | 27.11 | 4.84 | 2.01 |
| C | 35.96 | 31.42 | 19.96 | H | 24.86 | 3.31 | 1.72 |
| C | 35.86 | 30.01 | 19.99 | H | 24.19 | 22.68 | 2.23 |
| C | 37.03 | 29.21 | 0.01 | H | 26.07 | 20.75 | 2.33 |
| C | 40.13 | 4.85 | 0.01 | H | 0.85 | 20.02 | 1.65 |
| C | 39.98 | 5.56 | 1.23 | H | 3.30 | 21.03 | 1.73 |
| C | 39.57 | 6.92 | 1.23 | H | 4.53 | 22.34 | 3.94 |
| C | 39.33 | 7.58 | 20.00 | H | 6.92 | 21.55 | 3.90 |
| C | 39.57 | 6.91 | 18.78 | H | 8.42 | 22.20 | 1.98 |
| C | 39.97 | 5.55 | 18.78 | H | 7.53 | 23.65 | 0.13 |
| C | 4.82 | 34.63 | 19.81 | H | 5.15 | 24.45 | 0.19 |
| C | 5.45 | 34.77 | 18.55 | H | 0.18 | 5.07 | 0.09 |
| C | 6.82 | 0.20 | 18.49 | H | 2.06 | 4.61 | 3.99 |
| C | 7.54 | 0.40 | 19.69 | H | 2.67 | 7.05 | 3.98 |
| C | 6.94 | 0.16 | 0.95 | H | 2.09 | 8.48 | 2.01 |
| C | 5.58 | 34.72 | 1.01 | H | 0.80 | 7.50 | 0.07 |
| C | 35.38 | 34.50 | 19.82 | H | 23.57 | 24.54 | 0.14 |
| C | 34.61 | 34.48 | 1.01 | H | 21.10 | 24.92 | 0.17 |
| C | 33.19 | 34.53 | 0.95 | H | 20.05 | 1.26 | 2.07 |
| C | 32.54 | 34.57 | 19.70 | H | 21.50 | 2.14 | 3.93 |
| C | 33.30 | 34.56 | 18.50 | H | 23.98 | 1.79 | 3.87 |
| C | 34.71 | 34.52 | 18.56 | H | 23.81 | 13.70 | 2.45 |
| C | 12.59 | 2.72 | 19.93 | H | 25.20 | 11.34 | 2.45 |
| C | 11.31 | 3.44 | 19.96 | H | 1.66 | 10.19 | 1.51 |
| C | 11.61 | 4.85 | 19.98 | H | 3.94 | 11.71 | 1.53 |
| C | 13.09 | 4.94 | 19.98 | H | 4.61 | 17.26 | 2.24 |
| C | 13.86 | 6.13 | 0.00 | H | 2.72 | 19.18 | 2.33 |
| C | 15.28 | 6.23 | 19.99 | H | 27.95 | 19.92 | 1.65 |
| C | 16.08 | 7.47 | 19.99 | H | 25.50 | 18.91 | 1.72 |
| C | 17.45 | 7.07 | 19.95 | H | 24.28 | 17.59 | 3.93 |
| C | 17.46 | 5.58 | 19.93 | H | 21.89 | 18.38 | 3.90 |
| C | 18.60 | 4.74 | 19.89 | H | 20.38 | 17.74 | 1.98 |
| C | 18.53 | 3.31 | 19.93 | H | 21.26 | 16.28 | 0.13 |
| C | 19.60 | 2.31 | 19.95 | H | 23.64 | 15.48 | 0.18 |
| C | 18.97 | 1.01 | 19.99 | H | 28.63 | 9.94 | 3.98 |
| C | 17.53 | 1.25 | 19.99 | H | 26.73 | 10.39 | 0.10 |
| C | 16.30 | 0.52 | 19.99 | H | 26.12 | 7.95 | 0.10 |
| C | 15.80 | 34.06 | 19.99 | H | 26.72 | 6.53 | 2.07 |
| C | 14.35 | 34.15 | 19.95 | H | 27.99 | 7.50 | 4.01 |
| C | 14.01 | 0.67 | 19.94 | H | 5.22 | 15.40 | 0.15 |
| C | 12.74 | 1.31 | 19.89 | H | 7.69 | 15.02 | 0.17 |
| C | 9.97 | 2.99 | 19.99 | H | 8.76 | 13.74 | 2.06 |
| C | 10.53 | 5.78 | 0.00 | H | 7.32 | 12.85 | 3.93 |
| C | 9.20 | 5.31 | 0.01 | H | 4.83 | 13.20 | 3.88 |
| C | 8.92 | 3.92 | 0.01 | Cu | 0.02 | 0.01 | 2.00 |
| C | 15.74 | 8.86 | 0.01 | Cu | 28.79 | 14.98 | 2.00 |
| C | 18.49 | 8.05 | 19.95 | Au | 0.00 | 0.00 | 17.60 |
| C | 18.13 | 9.42 | 19.98 | Au | 2.88 | 0.00 | 17.60 |
| C | 16.77 | 9.82 | 0.01 | Au | 5.76 | 0.00 | 17.60 |
| C | 21.02 | 2.45 | 19.96 | Au | 8.64 | 0.00 | 17.60 |
| C | 19.79 | 34.76 | 0.01 | Au | 11.52 | 0.00 | 17.60 |
| C | 21.20 | 34.91 | 0.01 | Au | 14.40 | 0.00 | 17.60 |
| C | 21.81 | 1.28 | 19.99 | Au | 17.28 | 0.00 | 17.60 |
| C | 16.40 | 32.77 | 0.01 | Au | 20.16 | 0.00 | 17.60 |
| C | 13.54 | 32.98 | 19.95 | Au | 23.04 | 0.00 | 17.60 |
| C | 14.16 | 31.72 | 19.98 | Au | 25.92 | 0.00 | 17.60 |
| C | 15.58 | 31.62 | 0.01 | Au | 27.36 | 2.49 | 17.60 |
| C | 13.05 | 7.39 | 0.01 | Au | 1.44 | 2.49 | 17.60 |
| C | 12.60 | 7.93 | 1.24 | Au | 4.32 | 2.49 | 17.60 |
| C | 11.65 | 8.97 | 1.23 | Au | 7.20 | 2.49 | 17.60 |
| C | 11.18 | 9.46 | 19.99 | Au | 10.08 | 2.49 | 17.60 |
| C | 11.65 | 8.95 | 18.76 | Au | 12.96 | 2.49 | 17.60 |
| C | 12.60 | 7.91 | 18.77 | Au | 15.84 | 2.49 | 17.60 |
| C | 19.95 | 5.41 | 19.81 | Au | 18.72 | 2.49 | 17.60 |
| C | 20.53 | 5.75 | 18.56 | Au | 21.60 | 2.49 | 17.60 |
| C | 21.74 | 6.48 | 18.51 | Au | 24.48 | 2.49 | 17.60 |
| C | 22.39 | 6.87 | 19.70 | Au | 0.00 | 4.99 | 17.60 |
| C | 21.83 | 6.51 | 0.96 | Au | 2.88 | 4.99 | 17.60 |
| C | 20.63 | 5.78 | 1.01 | Au | 5.76 | 4.99 | 17.60 |
| C | 11.55 | 0.38 | 19.81 | Au | 8.64 | 4.99 | 17.60 |
| C | 10.99 | 34.76 | 1.01 | Au | 11.52 | 4.99 | 17.60 |
| C | 10.02 | 33.74 | 0.95 | Au | 14.40 | 4.99 | 17.60 |
| C | 9.59 | 33.23 | 19.70 | Au | 17.28 | 4.99 | 17.60 |
| C | 10.11 | 33.79 | 18.50 | Au | 20.16 | 4.99 | 17.60 |
| C | 11.07 | 34.82 | 18.56 | Au | 23.04 | 4.99 | 17.60 |
| N | 5.52 | 13.19 | 19.95 | Au | 25.92 | 4.99 | 17.60 |
| N | 2.98 | 14.63 | 19.97 | Au | 27.36 | 7.48 | 17.60 |
| N | 4.56 | 16.91 | 19.96 | Au | 1.44 | 7.48 | 17.60 |
| N | 6.72 | 15.68 | 19.98 | Au | 4.32 | 7.48 | 17.60 |
| N | 38.80 | 1.30 | 19.95 | Au | 7.20 | 7.48 | 17.60 |
| N | 1.37 | 1.32 | 19.97 | Au | 10.08 | 7.48 | 17.60 |
| N | 1.19 | 33.46 | 19.96 | Au | 12.96 | 7.48 | 17.60 |
| N | 39.04 | 33.44 | 19.97 | Au | 15.84 | 7.48 | 17.60 |
| N | 13.63 | 3.66 | 19.95 | Au | 18.72 | 7.48 | 17.60 |
| N | 16.13 | 5.14 | 19.97 | Au | 21.60 | 7.48 | 17.60 |
| N | 17.33 | 2.63 | 19.96 | Au | 24.48 | 7.48 | 17.60 |
| N | 15.19 | 1.37 | 19.98 | Au | 0.00 | 9.98 | 17.60 |
| H | 8.93 | 10.71 | 19.94 | Au | 2.88 | 9.98 | 17.60 |
| H | 4.21 | 9.04 | 0.03 | Au | 5.76 | 9.98 | 17.60 |
| H | 6.07 | 7.45 | 0.03 | Au | 8.64 | 9.98 | 17.60 |
| H | 8.47 | 8.25 | 0.00 | Au | 11.52 | 9.98 | 17.60 |
| H | 0.20 | 11.36 | 0.03 | Au | 14.40 | 9.98 | 17.60 |
| H | 39.31 | 16.26 | 19.92 | Au | 17.28 | 9.98 | 17.60 |
| H | 37.54 | 14.53 | 19.99 | Au | 20.16 | 9.98 | 17.60 |
| H | 38.13 | 12.09 | 0.02 | Au | 23.04 | 9.98 | 17.60 |
| H | 1.80 | 20.11 | 19.94 | Au | 25.92 | 9.98 | 17.60 |
| H | 6.84 | 20.56 | 0.03 | Au | 27.36 | 12.47 | 17.60 |
| H | 5.38 | 22.58 | 0.03 | Au | 1.44 | 12.47 | 17.60 |
| H | 2.88 | 22.37 | 20.00 | Au | 4.32 | 12.47 | 17.60 |
| H | 8.72 | 19.50 | 0.03 | Au | 7.20 | 12.47 | 17.60 |
| H | 10.90 | 14.93 | 19.92 | Au | 10.08 | 12.47 | 17.60 |
| H | 12.28 | 16.98 | 19.98 | Au | 12.96 | 12.47 | 17.60 |
| H | 11.20 | 19.25 | 0.03 | Au | 15.84 | 12.47 | 17.60 |
| H | 2.53 | 10.70 | 2.18 | Au | 18.72 | 12.47 | 17.60 |
| H | 1.87 | 8.24 | 2.18 | Au | 21.60 | 12.47 | 17.60 |
| H | 1.62 | 7.06 | 19.99 | Au | 24.48 | 12.47 | 17.60 |
| H | 1.88 | 8.25 | 17.82 | Au | 0.00 | 14.96 | 17.60 |
| H | 2.53 | 10.70 | 17.83 | Au | 2.88 | 14.96 | 17.60 |
| H | 0.76 | 17.81 | 17.63 | Au | 5.76 | 14.96 | 17.60 |
| H | 38.89 | 19.04 | 17.53 | Au | 8.64 | 14.96 | 17.60 |
| H | 37.71 | 19.69 | 19.66 | Au | 11.52 | 14.96 | 17.60 |
| H | 38.76 | 19.16 | 1.88 | Au | 14.40 | 14.96 | 17.60 |
| H | 0.64 | 17.94 | 1.97 | Au | 17.28 | 14.96 | 17.60 |
| H | 9.64 | 12.94 | 1.98 | Au | 20.16 | 14.96 | 17.60 |
| H | 12.06 | 12.27 | 1.88 | Au | 23.04 | 14.96 | 17.60 |
| H | 13.20 | 11.96 | 19.66 | Au | 25.92 | 14.96 | 17.60 |
| H | 11.88 | 12.27 | 17.53 | Au | 27.36 | 17.46 | 17.60 |
| H | 9.46 | 12.95 | 17.63 | Au | 1.44 | 17.46 | 17.60 |
| H | 34.51 | 1.80 | 19.92 | Au | 4.32 | 17.46 | 17.60 |
| H | 37.87 | 5.53 | 0.03 | Au | 7.20 | 17.46 | 17.60 |
| H | 35.48 | 6.02 | 0.02 | Au | 10.08 | 17.46 | 17.60 |
| H | 33.78 | 4.15 | 19.98 | Au | 12.96 | 17.46 | 17.60 |
| H | 2.10 | 5.49 | 0.05 | Au | 15.84 | 17.46 | 17.60 |
| H | 5.67 | 1.97 | 19.90 | Au | 18.72 | 17.46 | 17.60 |
| H | 6.27 | 4.35 | 19.96 | Au | 21.60 | 17.46 | 17.60 |
| H | 4.51 | 6.14 | 0.03 | Au | 24.48 | 17.46 | 17.60 |
| H | 5.20 | 32.12 | 19.95 | Au | 0.00 | 19.95 | 17.60 |
| H | 1.09 | 29.15 | 0.03 | Au | 2.88 | 19.95 | 17.60 |
| H | 3.39 | 28.16 | 0.02 | Au | 5.76 | 19.95 | 17.60 |
| H | 5.43 | 29.62 | 20.00 | Au | 8.64 | 19.95 | 17.60 |
| H | 39.21 | 29.13 | 0.03 | Au | 11.52 | 19.95 | 17.60 |
| H | 35.05 | 32.04 | 19.94 | Au | 14.40 | 19.95 | 17.60 |
| H | 34.87 | 29.54 | 19.99 | Au | 17.28 | 19.95 | 17.60 |
| H | 36.93 | 28.11 | 0.02 | Au | 20.16 | 19.95 | 17.60 |
| H | 40.12 | 5.02 | 2.18 | Au | 23.04 | 19.95 | 17.60 |
| H | 39.44 | 7.45 | 2.18 | Au | 25.92 | 19.95 | 17.60 |
| H | 39.04 | 8.64 | 20.00 | Au | 27.36 | 22.45 | 17.60 |
| H | 39.44 | 7.44 | 17.82 | Au | 1.44 | 22.45 | 17.60 |
| H | 40.12 | 5.01 | 17.84 | Au | 4.32 | 22.45 | 17.60 |
| H | 4.86 | 34.68 | 17.63 | Au | 7.20 | 22.45 | 17.60 |
| H | 7.31 | 0.37 | 17.52 | Au | 10.08 | 22.45 | 17.60 |
| H | 8.57 | 0.74 | 19.63 | Au | 12.96 | 22.45 | 17.60 |
| H | 7.52 | 0.31 | 1.87 | Au | 15.84 | 22.45 | 17.60 |
| H | 5.07 | 34.60 | 1.97 | Au | 18.72 | 22.45 | 17.60 |
| H | 35.13 | 34.49 | 1.98 | Au | 21.60 | 22.45 | 17.60 |
| H | 32.61 | 34.55 | 1.88 | Au | 24.48 | 22.45 | 17.60 |
| H | 31.44 | 34.63 | 19.65 | Au | 1.44 | 0.83 | 15.25 |
| H | 32.80 | 34.60 | 17.53 | Au | 4.32 | 0.83 | 15.25 |
| H | 35.31 | 34.55 | 17.64 | Au | 7.20 | 0.83 | 15.25 |
| H | 9.76 | 1.93 | 0.01 | Au | 10.08 | 0.83 | 15.25 |
| H | 10.69 | 6.86 | 0.02 | Au | 12.96 | 0.83 | 15.25 |
| H | 8.38 | 6.04 | 0.02 | Au | 15.84 | 0.83 | 15.25 |
| H | 7.89 | 3.55 | 0.03 | Au | 18.72 | 0.83 | 15.25 |
| H | 14.70 | 9.19 | 0.03 | Au | 21.60 | 0.83 | 15.25 |
| H | 19.54 | 7.78 | 19.93 | Au | 24.48 | 0.83 | 15.25 |
| H | 18.93 | 10.17 | 19.99 | Au | 27.36 | 0.83 | 15.25 |
| H | 16.52 | 10.89 | 0.03 | Au | 0.00 | 3.33 | 15.25 |
| H | 21.48 | 3.44 | 19.94 | Au | 2.88 | 3.33 | 15.25 |
| H | 19.35 | 33.76 | 0.03 | Au | 5.76 | 3.33 | 15.25 |
| H | 21.84 | 34.02 | 0.03 | Au | 8.64 | 3.33 | 15.25 |
| H | 22.90 | 1.37 | 20.00 | Au | 11.52 | 3.33 | 15.25 |
| H | 17.49 | 32.65 | 0.03 | Au | 14.40 | 3.33 | 15.25 |
| H | 12.45 | 33.04 | 19.93 | Au | 17.28 | 3.33 | 15.25 |
| H | 13.53 | 30.82 | 19.99 | Au | 20.16 | 3.33 | 15.25 |
| H | 16.04 | 30.62 | 0.02 | Au | 23.04 | 3.33 | 15.25 |
| H | 12.96 | 7.50 | 2.18 | Au | 25.92 | 3.33 | 15.25 |
| H | 11.26 | 9.39 | 2.17 | Au | 1.44 | 5.82 | 15.25 |
| H | 10.45 | 10.27 | 19.98 | Au | 4.32 | 5.82 | 15.25 |
| H | 11.27 | 9.35 | 17.81 | Au | 7.20 | 5.82 | 15.25 |
| H | 12.96 | 7.47 | 17.83 | Au | 10.08 | 5.82 | 15.25 |
| H | 20.00 | 5.47 | 17.63 | Au | 12.96 | 5.82 | 15.25 |
| H | 22.16 | 6.77 | 17.53 | Au | 15.84 | 5.82 | 15.25 |
| H | 23.32 | 7.46 | 19.66 | Au | 18.72 | 5.82 | 15.25 |
| H | 22.33 | 6.82 | 1.89 | Au | 21.60 | 5.82 | 15.25 |
| H | 20.17 | 5.52 | 1.97 | Au | 24.48 | 5.82 | 15.25 |
| H | 11.34 | 0.23 | 1.97 | Au | 27.36 | 5.82 | 15.25 |
| H | 9.58 | 33.35 | 1.88 | Au | 0.00 | 8.31 | 15.25 |
| H | 8.81 | 32.46 | 19.65 | Au | 2.88 | 8.31 | 15.25 |
| H | 9.73 | 33.45 | 17.53 | Au | 5.76 | 8.31 | 15.25 |
| H | 11.48 | 0.33 | 17.63 | Au | 8.64 | 8.31 | 15.25 |
| Cu | 4.89 | 15.02 | 19.96 | Au | 11.52 | 8.31 | 15.25 |
| Cu | 40.26 | 0.01 | 19.96 | Au | 14.40 | 8.31 | 15.25 |
| Cu | 15.53 | 3.28 | 19.96 | Au | 17.28 | 8.31 | 15.25 |
|  |  |  |  | Au | 20.16 | 8.31 | 15.25 |
|  |  |  |  | Au | 23.04 | 8.31 | 15.25 |
|  |  |  |  | Au | 25.92 | 8.31 | 15.25 |
|  |  |  |  | Au | 1.44 | 10.81 | 15.25 |
|  |  |  |  | Au | 4.32 | 10.81 | 15.25 |
|  |  |  |  | Au | 7.20 | 10.81 | 15.25 |
|  |  |  |  | Au | 10.08 | 10.81 | 15.25 |
|  |  |  |  | Au | 12.96 | 10.81 | 15.25 |
|  |  |  |  | Au | 15.84 | 10.81 | 15.25 |
|  |  |  |  | Au | 18.72 | 10.81 | 15.25 |
|  |  |  |  | Au | 21.60 | 10.81 | 15.25 |
|  |  |  |  | Au | 24.48 | 10.81 | 15.25 |
|  |  |  |  | Au | 27.36 | 10.81 | 15.25 |
|  |  |  |  | Au | 0.00 | 13.30 | 15.25 |
|  |  |  |  | Au | 2.88 | 13.30 | 15.25 |
|  |  |  |  | Au | 5.76 | 13.30 | 15.25 |
|  |  |  |  | Au | 8.64 | 13.30 | 15.25 |
|  |  |  |  | Au | 11.52 | 13.30 | 15.25 |
|  |  |  |  | Au | 14.40 | 13.30 | 15.25 |
|  |  |  |  | Au | 17.28 | 13.30 | 15.25 |
|  |  |  |  | Au | 20.16 | 13.30 | 15.25 |
|  |  |  |  | Au | 23.04 | 13.30 | 15.25 |
|  |  |  |  | Au | 25.92 | 13.30 | 15.25 |
|  |  |  |  | Au | 1.44 | 15.80 | 15.25 |
|  |  |  |  | Au | 4.32 | 15.80 | 15.25 |
|  |  |  |  | Au | 7.20 | 15.80 | 15.25 |
|  |  |  |  | Au | 10.08 | 15.80 | 15.25 |
|  |  |  |  | Au | 12.96 | 15.80 | 15.25 |
|  |  |  |  | Au | 15.84 | 15.80 | 15.25 |
|  |  |  |  | Au | 18.72 | 15.80 | 15.25 |
|  |  |  |  | Au | 21.60 | 15.80 | 15.25 |
|  |  |  |  | Au | 24.48 | 15.80 | 15.25 |
|  |  |  |  | Au | 27.36 | 15.80 | 15.25 |
|  |  |  |  | Au | 0.00 | 18.29 | 15.25 |
|  |  |  |  | Au | 2.88 | 18.29 | 15.25 |
|  |  |  |  | Au | 5.76 | 18.29 | 15.25 |
|  |  |  |  | Au | 8.64 | 18.29 | 15.25 |
|  |  |  |  | Au | 11.52 | 18.29 | 15.25 |
|  |  |  |  | Au | 14.40 | 18.29 | 15.25 |
|  |  |  |  | Au | 17.28 | 18.29 | 15.25 |
|  |  |  |  | Au | 20.16 | 18.29 | 15.25 |
|  |  |  |  | Au | 23.04 | 18.29 | 15.25 |
|  |  |  |  | Au | 25.92 | 18.29 | 15.25 |
|  |  |  |  | Au | 1.44 | 20.78 | 15.25 |
|  |  |  |  | Au | 4.32 | 20.78 | 15.25 |
|  |  |  |  | Au | 7.20 | 20.78 | 15.25 |
|  |  |  |  | Au | 10.08 | 20.78 | 15.25 |
|  |  |  |  | Au | 12.96 | 20.78 | 15.25 |
|  |  |  |  | Au | 15.84 | 20.78 | 15.25 |
|  |  |  |  | Au | 18.72 | 20.78 | 15.25 |
|  |  |  |  | Au | 21.60 | 20.78 | 15.25 |
|  |  |  |  | Au | 24.48 | 20.78 | 15.25 |
|  |  |  |  | Au | 27.36 | 20.78 | 15.25 |
|  |  |  |  | Au | 0.00 | 23.28 | 15.25 |
|  |  |  |  | Au | 2.88 | 23.28 | 15.25 |
|  |  |  |  | Au | 5.76 | 23.28 | 15.25 |
|  |  |  |  | Au | 8.64 | 23.28 | 15.25 |
|  |  |  |  | Au | 11.52 | 23.28 | 15.25 |
|  |  |  |  | Au | 14.40 | 23.28 | 15.25 |
|  |  |  |  | Au | 17.28 | 23.28 | 15.25 |
|  |  |  |  | Au | 20.16 | 23.28 | 15.25 |
|  |  |  |  | Au | 23.04 | 23.28 | 15.25 |
|  |  |  |  | Au | 25.92 | 23.28 | 15.25 |
|  |  |  |  | Au | 0.00 | 1.66 | 12.90 |
|  |  |  |  | Au | 2.88 | 1.66 | 12.90 |
|  |  |  |  | Au | 5.76 | 1.66 | 12.90 |
|  |  |  |  | Au | 8.64 | 1.66 | 12.90 |
|  |  |  |  | Au | 11.52 | 1.66 | 12.90 |
|  |  |  |  | Au | 14.40 | 1.66 | 12.90 |
|  |  |  |  | Au | 17.28 | 1.66 | 12.90 |
|  |  |  |  | Au | 20.16 | 1.66 | 12.90 |
|  |  |  |  | Au | 23.04 | 1.66 | 12.90 |
|  |  |  |  | Au | 25.92 | 1.66 | 12.90 |
|  |  |  |  | Au | 27.36 | 4.16 | 12.90 |
|  |  |  |  | Au | 1.44 | 4.16 | 12.90 |
|  |  |  |  | Au | 4.32 | 4.16 | 12.90 |
|  |  |  |  | Au | 7.20 | 4.16 | 12.90 |
|  |  |  |  | Au | 10.08 | 4.16 | 12.90 |
|  |  |  |  | Au | 12.96 | 4.16 | 12.90 |
|  |  |  |  | Au | 15.84 | 4.16 | 12.90 |
|  |  |  |  | Au | 18.72 | 4.16 | 12.90 |
|  |  |  |  | Au | 21.60 | 4.16 | 12.90 |
|  |  |  |  | Au | 24.48 | 4.16 | 12.90 |
|  |  |  |  | Au | 0.00 | 6.65 | 12.90 |
|  |  |  |  | Au | 2.88 | 6.65 | 12.90 |
|  |  |  |  | Au | 5.76 | 6.65 | 12.90 |
|  |  |  |  | Au | 8.64 | 6.65 | 12.90 |
|  |  |  |  | Au | 11.52 | 6.65 | 12.90 |
|  |  |  |  | Au | 14.40 | 6.65 | 12.90 |
|  |  |  |  | Au | 17.28 | 6.65 | 12.90 |
|  |  |  |  | Au | 20.16 | 6.65 | 12.90 |
|  |  |  |  | Au | 23.04 | 6.65 | 12.90 |
|  |  |  |  | Au | 25.92 | 6.65 | 12.90 |
|  |  |  |  | Au | 27.36 | 9.15 | 12.90 |
|  |  |  |  | Au | 1.44 | 9.15 | 12.90 |
|  |  |  |  | Au | 4.32 | 9.15 | 12.90 |
|  |  |  |  | Au | 7.20 | 9.15 | 12.90 |
|  |  |  |  | Au | 10.08 | 9.15 | 12.90 |
|  |  |  |  | Au | 12.96 | 9.15 | 12.90 |
|  |  |  |  | Au | 15.84 | 9.15 | 12.90 |
|  |  |  |  | Au | 18.72 | 9.15 | 12.90 |
|  |  |  |  | Au | 21.60 | 9.15 | 12.90 |
|  |  |  |  | Au | 24.48 | 9.15 | 12.90 |
|  |  |  |  | Au | 0.00 | 11.64 | 12.90 |
|  |  |  |  | Au | 2.88 | 11.64 | 12.90 |
|  |  |  |  | Au | 5.76 | 11.64 | 12.90 |
|  |  |  |  | Au | 8.64 | 11.64 | 12.90 |
|  |  |  |  | Au | 11.52 | 11.64 | 12.90 |
|  |  |  |  | Au | 14.40 | 11.64 | 12.90 |
|  |  |  |  | Au | 17.28 | 11.64 | 12.90 |
|  |  |  |  | Au | 20.16 | 11.64 | 12.90 |
|  |  |  |  | Au | 23.04 | 11.64 | 12.90 |
|  |  |  |  | Au | 25.92 | 11.64 | 12.90 |
|  |  |  |  | Au | 27.36 | 14.13 | 12.90 |
|  |  |  |  | Au | 1.44 | 14.13 | 12.90 |
|  |  |  |  | Au | 4.32 | 14.13 | 12.90 |
|  |  |  |  | Au | 7.20 | 14.13 | 12.90 |
|  |  |  |  | Au | 10.08 | 14.13 | 12.90 |
|  |  |  |  | Au | 12.96 | 14.13 | 12.90 |
|  |  |  |  | Au | 15.84 | 14.13 | 12.90 |
|  |  |  |  | Au | 18.72 | 14.13 | 12.90 |
|  |  |  |  | Au | 21.60 | 14.13 | 12.90 |
|  |  |  |  | Au | 24.48 | 14.13 | 12.90 |
|  |  |  |  | Au | 0.00 | 16.63 | 12.90 |
|  |  |  |  | Au | 2.88 | 16.63 | 12.90 |
|  |  |  |  | Au | 5.76 | 16.63 | 12.90 |
|  |  |  |  | Au | 8.64 | 16.63 | 12.90 |
|  |  |  |  | Au | 11.52 | 16.63 | 12.90 |
|  |  |  |  | Au | 14.40 | 16.63 | 12.90 |
|  |  |  |  | Au | 17.28 | 16.63 | 12.90 |
|  |  |  |  | Au | 20.16 | 16.63 | 12.90 |
|  |  |  |  | Au | 23.04 | 16.63 | 12.90 |
|  |  |  |  | Au | 25.92 | 16.63 | 12.90 |
|  |  |  |  | Au | 27.36 | 19.12 | 12.90 |
|  |  |  |  | Au | 1.44 | 19.12 | 12.90 |
|  |  |  |  | Au | 4.32 | 19.12 | 12.90 |
|  |  |  |  | Au | 7.20 | 19.12 | 12.90 |
|  |  |  |  | Au | 10.08 | 19.12 | 12.90 |
|  |  |  |  | Au | 12.96 | 19.12 | 12.90 |
|  |  |  |  | Au | 15.84 | 19.12 | 12.90 |
|  |  |  |  | Au | 18.72 | 19.12 | 12.90 |
|  |  |  |  | Au | 21.60 | 19.12 | 12.90 |
|  |  |  |  | Au | 24.48 | 19.12 | 12.90 |
|  |  |  |  | Au | 0.00 | 21.62 | 12.90 |
|  |  |  |  | Au | 2.88 | 21.62 | 12.90 |
|  |  |  |  | Au | 5.76 | 21.62 | 12.90 |
|  |  |  |  | Au | 8.64 | 21.62 | 12.90 |
|  |  |  |  | Au | 11.52 | 21.62 | 12.90 |
|  |  |  |  | Au | 14.40 | 21.62 | 12.90 |
|  |  |  |  | Au | 17.28 | 21.62 | 12.90 |
|  |  |  |  | Au | 20.16 | 21.62 | 12.90 |
|  |  |  |  | Au | 23.04 | 21.62 | 12.90 |
|  |  |  |  | Au | 25.92 | 21.62 | 12.90 |
|  |  |  |  | Au | 27.36 | 24.11 | 12.90 |
|  |  |  |  | Au | 1.44 | 24.11 | 12.90 |
|  |  |  |  | Au | 4.32 | 24.11 | 12.90 |
|  |  |  |  | Au | 7.20 | 24.11 | 12.90 |
|  |  |  |  | Au | 10.08 | 24.11 | 12.90 |
|  |  |  |  | Au | 12.96 | 24.11 | 12.90 |
|  |  |  |  | Au | 15.84 | 24.11 | 12.90 |
|  |  |  |  | Au | 18.72 | 24.11 | 12.90 |
|  |  |  |  | Au | 21.60 | 24.11 | 12.90 |
|  |  |  |  | Au | 24.48 | 24.11 | 12.90 |
