# Supplementary material for: Modulation of the molecular spintronic properties of adsorbed copper corroles
Source: Nat Commun. 2015 Jun 26;6:7547. doi: 10.1038/ncomms8547 (PMC4491828; doi:10.1038/ncomms8547)
Supplement: Supplementary Data 4 — Coordinates for the B3LYP optimized structures. [file ncomms8547-s5.docx]

**Coordinates for the B3LYP optimized structures.**

Coordinates for the B3LYP/6-31G(d) geometries calculated for the singlet and triplet groundstates of **Cu-TPC** are provided below in Å.

| **Cu-TPC** singlet groundstate | | | | **Cu-TPC** triplet groundstate | | | |
| --- | --- | --- | --- | --- | --- | --- | --- |
|  | *x* | *y* | *z* |  | *x* | *y* | *z* |
| N | 1.215 | −2.091 | −0.244 | N | 1.236 | −2.112 | −0.179 |
| N | −1.410 | 0.580 | −0.152 | N | −1.433 | 0.612 | −0.033 |
| Cu | 0.001 | −0.674 | −0.014 | Cu | −0.003 | −0.658 | −0.119 |
| N | 1.414 | 0.572 | 0.146 | N | 1.434 | 0.601 | 0.023 |
| C | 3.363 | −0.989 | −0.024 | C | 3.363 | −0.995 | −0.006 |
| C | −3.364 | −0.962 | 0.054 | C | −3.372 | −0.969 | 0.005 |
| C | 0.008 | 2.607 | −0.048 | C | 0.008 | 2.620 | −0.060 |
| C | −1.804 | −4.254 | −0.097 | C | −1.811 | −4.286 | 0.012 |
| C | −2.968 | −3.510 | −0.051 | C | −2.982 | −3.547 | 0.050 |
| C | −0.721 | −3.336 | 0.012 | C | −0.740 | −3.348 | −0.120 |
| C | −2.596 | −2.129 | 0.072 | C | −2.616 | −2.155 | −0.068 |
| C | 2.520 | 2.526 | −0.335 | C | 2.567 | 2.576 | −0.154 |
| C | 3.449 | 1.522 | −0.323 | C | 3.495 | 1.564 | −0.134 |
| C | 1.244 | 1.934 | −0.063 | C | 1.271 | 1.963 | −0.050 |
| C | 2.764 | 0.294 | −0.044 | C | 2.779 | 0.322 | −0.021 |
| N | −1.225 | −2.078 | 0.188 | N | −1.255 | −2.102 | −0.181 |
| C | 2.581 | −2.151 | −0.065 | C | 2.597 | −2.175 | −0.062 |
| C | 2.931 | −3.527 | 0.142 | C | 2.951 | −3.570 | 0.063 |
| C | 1.761 | −4.262 | 0.155 | C | 1.775 | −4.299 | 0.022 |
| C | 0.695 | −3.342 | −0.038 | C | 0.712 | −3.353 | −0.117 |
| C | −2.513 | 2.560 | 0.209 | C | −2.555 | 2.600 | −0.020 |
| C | −3.447 | 1.562 | 0.230 | C | −3.490 | 1.594 | −0.008 |
| C | −2.761 | 0.317 | 0.044 | C | −2.781 | 0.343 | −0.004 |
| C | −1.234 | 1.948 | −0.009 | C | −1.261 | 1.975 | −0.047 |
| C | 0.016 | 4.098 | −0.080 | C | 0.016 | 4.112 | −0.097 |
| C | 0.699 | 4.834 | 0.903 | C | 0.601 | 4.852 | 0.945 |
| C | 0.704 | 6.228 | 0.876 | C | 0.607 | 6.246 | 0.914 |
| C | 0.032 | 6.913 | −0.139 | C | 0.036 | 6.927 | −0.163 |
| C | −0.648 | 6.194 | −1.124 | C | −0.544 | 6.204 | −1.207 |
| C | −0.658 | 4.800 | −1.094 | C | −0.557 | 4.810 | −1.173 |
| C | −4.854 | −1.078 | 0.069 | C | −4.851 | −1.086 | 0.112 |
| C | −5.592 | −0.753 | 1.218 | C | −5.554 | −0.496 | 1.178 |
| C | −6.982 | −0.879 | 1.230 | C | −6.937 | −0.634 | 1.283 |
| C | −7.656 | −1.335 | 0.096 | C | −7.647 | −1.359 | 0.324 |
| C | −6.932 | −1.666 | −1.050 | C | −6.962 | −1.950 | −0.740 |
| C | −5.542 | −1.537 | −1.065 | C | −5.578 | −1.818 | −0.843 |
| C | 4.843 | −1.108 | 0.022 | C | 4.842 | −1.116 | 0.074 |
| C | 5.594 | −0.422 | 0.992 | C | 5.570 | −0.483 | 1.097 |
| C | 6.981 | −0.549 | 1.044 | C | 6.955 | −0.623 | 1.179 |
| C | 7.647 | −1.363 | 0.125 | C | 7.641 | −1.392 | 0.237 |
| C | 6.915 | −2.049 | −0.846 | C | 6.932 | −2.025 | −0.786 |
| C | 5.527 | −1.925 | −0.896 | C | 5.546 | −1.891 | −0.865 |
| H | −1.718 | −5.328 | −0.205 | H | −1.718 | −5.362 | 0.086 |
| H | −3.981 | −3.874 | −0.147 | H | −3.986 | −3.930 | 0.170 |
| H | 2.683 | 3.571 | −0.556 | H | 2.758 | 3.635 | −0.251 |
| H | 4.507 | 1.603 | −0.526 | H | 4.567 | 1.664 | −0.217 |
| H | 3.933 | −3.892 | 0.323 | H | 3.951 | −3.961 | 0.188 |
| H | 1.659 | −5.328 | 0.314 | H | 1.673 | −5.374 | 0.099 |
| H | −2.674 | 3.618 | 0.361 | H | −2.742 | 3.664 | −0.009 |
| H | −4.513 | 1.662 | 0.379 | H | −4.564 | 1.707 | −0.008 |
| H | 1.215 | 4.304 | 1.698 | H | 1.040 | 4.324 | 1.787 |
| H | 1.230 | 6.780 | 1.650 | H | 1.055 | 6.800 | 1.734 |
| H | −1.167 | 6.719 | −1.922 | H | −0.984 | 6.725 | −2.053 |
| H | −1.179 | 4.243 | −1.867 | H | −1.002 | 4.250 | −1.991 |
| H | −5.069 | −0.409 | 2.106 | H | −5.004 | 0.055 | 1.935 |
| H | −7.537 | −0.625 | 2.130 | H | −7.460 | −0.180 | 2.120 |
| H | −7.448 | −2.020 | −1.939 | H | −7.507 | −2.510 | −1.495 |
| H | −4.981 | −1.787 | −1.961 | H | −5.049 | −2.268 | −1.678 |
| H | 5.079 | 0.200 | 1.718 | H | 5.039 | 0.104 | 1.840 |
| H | 7.542 | −0.017 | 1.808 | H | 7.497 | −0.134 | 1.984 |
| H | 7.425 | −2.678 | −1.571 | H | 7.458 | −2.619 | −1.528 |
| H | 4.962 | −2.447 | −1.663 | H | 4.999 | −2.371 | −1.671 |
| H | −8.738 | −1.434 | 0.107 | H | −8.725 | −1.464 | 0.405 |
| H | 0.038 | 8.000 | −0.161 | H | 0.043 | 8.013 | −0.188 |
| H | 8.729 | −1.461 | 0.164 | H | 8.721 | −1.498 | 0.300 |

Coordinates for the B3LYP/6-31G(d) geometries calculated for the singlet and triplet groundstates of **Cu-BCOD** are provided below in Å.

| **Cu-BCOD** singlet groundstate | | | | **Cu-BCOD** triplet groundstate | | | |
| --- | --- | --- | --- | --- | --- | --- | --- |
|  | *x* | *y* | *z* |  | *x* | *y* | *z* |
| N | 1.251 | −1.692 | 0.223 | N | −1.243 | −1.720 | −0.134 |
| N | −1.410 | 0.994 | 0.141 | N | 1.439 | 1.017 | 0.019 |
| Cu | 0.014 | −0.273 | 0.008 | Cu | −0.004 | −0.258 | −0.029 |
| N | 1.442 | 0.981 | −0.182 | N | −1.454 | 1.008 | 0.027 |
| C | 3.383 | −0.587 | −0.075 | C | −3.369 | −0.597 | −0.030 |
| C | −3.355 | −0.569 | 0.079 | C | 3.364 | −0.576 | −0.015 |
| C | 0.021 | 3.001 | −0.051 | C | −0.014 | 3.015 | 0.074 |
| C | −1.754 | −3.833 | 0.415 | C | 1.811 | −3.892 | −0.186 |
| C | −2.926 | −3.099 | 0.430 | C | 2.984 | −3.156 | −0.158 |
| C | −0.698 | −2.939 | 0.104 | C | 0.730 | −2.963 | −0.109 |
| C | −2.593 | −1.737 | 0.115 | C | 2.616 | −1.760 | −0.062 |
| C | 2.524 | 2.920 | 0.418 | C | −2.588 | 2.991 | −0.025 |
| C | 3.459 | 1.915 | 0.388 | C | −3.518 | 1.974 | −0.061 |
| C | 1.257 | 2.343 | 0.070 | C | −1.284 | 2.378 | 0.025 |
| C | 2.791 | 0.697 | 0.030 | C | −2.805 | 0.725 | −0.023 |
| N | −1.226 | −1.698 | −0.140 | N | 1.244 | −1.714 | −0.035 |
| C | 2.614 | −1.752 | −0.047 | C | −2.614 | −1.777 | −0.066 |
| C | 2.940 | −3.132 | −0.282 | C | −2.972 | −3.179 | −0.024 |
| C | 0.716 | −2.944 | 0.055 | C | −0.720 | −2.968 | −0.112 |
| C | −2.478 | 2.912 | −0.543 | C | 2.560 | 3.002 | 0.179 |
| C | −3.417 | 1.912 | −0.487 | C | 3.497 | 1.991 | 0.148 |
| C | −2.759 | 0.707 | −0.071 | C | 2.792 | 0.741 | 0.044 |
| C | −1.219 | 2.346 | −0.154 | C | 1.260 | 2.384 | 0.089 |
| C | 0.028 | 4.494 | −0.074 | C | −0.018 | 4.513 | 0.105 |
| C | −0.537 | 5.228 | 0.980 | C | 0.086 | 5.249 | −1.083 |
| C | −0.522 | 6.624 | 0.964 | C | 0.081 | 6.645 | −1.056 |
| C | 0.048 | 7.306 | −0.114 | C | −0.026 | 7.322 | 0.160 |
| C | 0.609 | 6.585 | −1.171 | C | −0.130 | 6.596 | 1.349 |
| C | 0.605 | 5.189 | −1.147 | C | −0.128 | 5.200 | 1.321 |
| C | −4.838 | −0.674 | 0.187 | C | 4.854 | −0.713 | −0.034 |
| C | −5.510 | −0.151 | 1.304 | C | 5.557 | −0.664 | −1.247 |
| C | −6.897 | −0.256 | 1.413 | C | 6.944 | −0.814 | −1.269 |
| C | −7.636 | −0.876 | 0.403 | C | 7.648 | −1.017 | −0.080 |
| C | −6.979 | −1.398 | −0.714 | C | 6.956 | −1.072 | 1.132 |
| C | −5.590 | −1.302 | −0.817 | C | 5.568 | −0.924 | 1.154 |
| C | 4.863 | −0.698 | −0.196 | C | −4.857 | −0.742 | 0.020 |
| C | 5.621 | −1.342 | 0.795 | C | −5.587 | −1.020 | −1.145 |
| C | 7.008 | −1.438 | 0.680 | C | −6.974 | −1.173 | −1.095 |
| C | 7.659 | −0.903 | −0.434 | C | −7.648 | −1.061 | 0.123 |
| C | 6.915 | −0.267 | −1.430 | C | −6.928 | −0.795 | 1.290 |
| C | 5.529 | −0.161 | −1.310 | C | −5.543 | −0.634 | 1.239 |
| C | −1.919 | −5.288 | 0.772 | C | 2.018 | −5.385 | −0.251 |
| C | −4.135 | −3.914 | 0.835 | C | 4.231 | −4.008 | −0.194 |
| C | 3.048 | 4.228 | 0.974 | C | −3.196 | 4.375 | −0.128 |
| C | 4.821 | 2.325 | 0.908 | C | −4.946 | 2.454 | −0.213 |
| C | 4.118 | −3.953 | −0.762 | C | −4.205 | −4.037 | 0.134 |
| C | −3.014 | 4.228 | −1.066 | C | 3.165 | 4.388 | 0.263 |
| C | −4.796 | 2.336 | −0.946 | C | 4.929 | 2.480 | 0.201 |
| C | −4.642 | 2.975 | −2.324 | C | 5.055 | 3.417 | 1.397 |
| C | −3.727 | 3.945 | −2.385 | C | 4.148 | 4.396 | 1.430 |
| C | 4.322 | 4.576 | 0.212 | C | −4.303 | 4.479 | 0.917 |
| C | 5.232 | 3.598 | 0.178 | C | −5.203 | 3.493 | 0.872 |
| C | 4.207 | −5.188 | 0.131 | C | −4.157 | −5.121 | −0.940 |
| C | 3.073 | −5.888 | 0.231 | C | −3.019 | −5.820 | −0.979 |
| C | −2.663 | −5.347 | 2.105 | C | 2.972 | −5.672 | −1.407 |
| C | −3.797 | −4.640 | 2.135 | C | 4.106 | −4.965 | −1.377 |
| C | 1.767 | −3.861 | −0.204 | C | −1.794 | −3.907 | −0.058 |
| C | 1.905 | −5.317 | −0.567 | C | −1.984 | −5.399 | 0.061 |
| C | 3.514 | 3.894 | 2.442 | C | −3.934 | 4.400 | −1.521 |
| C | 4.571 | 2.758 | 2.403 | C | −4.974 | 3.250 | −1.573 |
| C | −2.924 | −5.869 | −0.292 | C | 2.827 | −5.750 | 1.053 |
| C | −4.247 | −5.052 | −0.251 | C | 4.144 | −4.925 | 1.088 |
| C | −5.214 | 3.511 | 0.014 | C | 5.104 | 3.396 | −1.070 |
| C | −4.153 | 4.644 | −0.061 | C | 4.051 | 4.536 | −1.031 |
| C | 2.387 | −5.316 | −2.068 | C | −2.685 | −5.618 | 1.456 |
| C | 3.702 | −4.495 | −2.183 | C | −4.006 | −4.800 | 1.501 |
| H | −0.980 | 4.697 | 1.818 | H | 0.170 | 4.722 | −2.030 |
| H | −0.954 | 7.178 | 1.792 | H | 0.161 | 7.203 | −1.986 |
| H | 1.051 | 7.109 | −2.014 | H | −0.214 | 7.116 | 2.300 |
| H | 1.048 | 4.628 | −1.965 | H | −0.211 | 4.635 | 2.246 |
| H | −4.934 | 0.330 | 2.089 | H | 5.009 | −0.508 | −2.172 |
| H | −7.401 | 0.146 | 2.288 | H | 7.476 | −0.775 | −2.217 |
| H | −7.547 | −1.878 | −1.506 | H | 7.496 | −1.231 | 2.061 |
| H | −5.079 | −1.704 | −1.688 | H | 5.029 | −0.966 | 2.096 |
| H | 5.113 | −1.758 | 1.661 | H | −5.060 | −1.112 | −2.091 |
| H | 7.581 | −1.930 | 1.462 | H | −7.526 | −1.381 | −2.007 |
| H | 7.413 | 0.147 | −2.302 | H | −7.446 | −0.711 | 2.243 |
| H | 4.950 | 0.339 | −2.081 | H | −4.984 | −0.421 | 2.146 |
| H | −8.717 | −0.953 | 0.486 | H | 8.728 | −1.132 | −0.098 |
| H | 0.055 | 8.392 | −0.130 | H | −0.029 | 8.409 | 0.182 |
| H | 8.739 | −0.980 | −0.524 | H | −8.727 | −1.183 | 0.163 |
| H | −0.983 | −5.851 | 0.784 | H | 1.094 | −5.962 | −0.313 |
| H | −5.061 | −3.345 | 0.899 | H | 5.162 | −3.442 | −0.205 |
| H | 2.327 | 5.042 | 0.978 | H | −2.484 | 5.192 | −0.059 |
| H | 5.579 | 1.547 | 0.857 | H | −5.692 | 1.664 | −0.219 |
| H | 5.057 | −3.405 | −0.818 | H | −5.142 | −3.482 | 0.133 |
| H | 0.985 | −5.895 | −0.453 | H | −1.058 | −5.974 | −0.005 |
| H | −2.270 | 5.017 | −1.156 | H | 2.441 | 5.197 | 0.327 |
| H | −5.540 | 1.543 | −0.936 | H | 5.677 | 1.692 | 0.211 |
| H | −5.274 | 2.671 | −3.154 | H | 5.856 | 3.294 | 2.121 |
| H | −3.517 | 4.538 | −3.272 | H | 4.112 | 5.178 | 2.184 |
| H | 4.462 | 5.562 | −0.222 | H | −4.350 | 5.325 | 1.597 |
| H | 6.211 | 3.686 | −0.285 | H | −6.080 | 3.430 | 1.511 |
| H | 5.144 | −5.473 | 0.600 | H | −5.016 | −5.313 | −1.578 |
| H | 2.972 | −6.815 | 0.789 | H | −2.834 | −6.655 | −1.649 |
| H | −2.295 | −5.955 | 2.926 | H | 2.740 | −6.429 | −2.152 |
| H | −4.472 | −4.604 | 2.986 | H | 4.913 | −5.072 | −2.096 |
| H | 2.641 | 3.600 | 3.035 | H | −3.191 | 4.299 | −2.319 |
| H | 3.929 | 4.801 | 2.896 | H | −4.419 | 5.374 | −1.645 |
| H | 5.520 | 3.089 | 2.839 | H | −5.985 | 3.641 | −1.732 |
| H | 4.234 | 1.886 | 2.974 | H | −4.757 | 2.558 | −2.395 |
| H | −2.465 | −5.820 | −1.285 | H | 2.207 | −5.536 | 1.930 |
| H | −3.110 | −6.925 | −0.069 | H | 3.039 | −6.825 | 1.053 |
| H | −5.095 | −5.698 | 0.001 | H | 5.018 | −5.586 | 1.108 |
| H | −4.457 | −4.598 | −1.225 | H | 4.189 | −4.294 | 1.981 |
| H | −6.201 | 3.879 | −0.286 | H | 6.121 | 3.802 | −1.076 |
| H | −5.306 | 3.123 | 1.034 | H | 4.990 | 2.783 | −1.970 |
| H | −3.710 | 4.836 | 0.922 | H | 3.401 | 4.504 | −1.913 |
| H | −4.604 | 5.581 | −0.405 | H | 4.536 | 5.518 | −1.017 |
| H | 2.541 | −6.351 | −2.394 | H | −2.882 | −6.687 | 1.593 |
| H | 1.599 | −4.886 | −2.696 | H | −2.002 | −5.304 | 2.252 |
| H | 4.515 | −5.115 | −2.577 | H | −4.866 | −5.457 | 1.666 |
| H | 3.576 | −3.647 | −2.864 | H | −3.988 | −4.070 | 2.316 |

Coordinates for the B3LYP/6-31G(d) geometries calculated for the singlet and triplet groundstates of **Cu-Benzo** are provided below in Å .

| **Cu-Benzo** singlet groundstate | | | | **Cu-Benzo** triplet groundstate | | | |
| --- | --- | --- | --- | --- | --- | --- | --- |
|  | *x* | *y* | *z* |  | *x* | *y* | *z* |
| N | 1.250 | −1.822 | 0.188 | N | 1.248 | −1.818 | −0.005 |
| N | −1.450 | 0.888 | 0.102 | N | −1.450 | 0.920 | 0.009 |
| Cu | 0.000 | −0.378 | 0.000 | Cu | 0.000 | −0.353 | 0.000 |
| N | 1.450 | 0.888 | −0.102 | N | 1.450 | 0.920 | −0.009 |
| C | 3.368 | −0.694 | −0.041 | C | 3.368 | −0.669 | 0.001 |
| C | −3.368 | −0.695 | 0.041 | C | −3.368 | −0.670 | −0.001 |
| C | 0.000 | 2.879 | 0.000 | C | 0.000 | 2.918 | 0.000 |
| C | −1.737 | −3.978 | 0.404 | C | −1.783 | −4.041 | −0.107 |
| C | −2.952 | −3.233 | 0.440 | C | −2.990 | −3.276 | −0.106 |
| C | −0.705 | −3.046 | 0.041 | C | −0.698 | −3.087 | −0.023 |
| C | −2.608 | −1.856 | 0.090 | C | −2.589 | −1.862 | −0.034 |
| C | 2.469 | 2.809 | 0.688 | C | 2.585 | 2.928 | −0.119 |
| C | 3.440 | 1.776 | 0.651 | C | 3.548 | 1.889 | −0.111 |
| C | 1.231 | 2.229 | 0.199 | C | 1.262 | 2.278 | −0.044 |
| C | 2.773 | 0.588 | 0.151 | C | 2.811 | 0.619 | −0.037 |
| N | −1.249 | −1.822 | −0.188 | N | −1.248 | −1.818 | 0.005 |
| C | 2.609 | −1.856 | −0.090 | C | 2.590 | −1.862 | 0.034 |
| C | 2.953 | −3.233 | −0.440 | C | 2.991 | −3.276 | 0.106 |
| C | 1.738 | −3.978 | −0.404 | C | 1.783 | −4.040 | 0.107 |
| C | 0.706 | −3.046 | −0.042 | C | 0.698 | −3.086 | 0.023 |
| C | −2.469 | 2.808 | −0.688 | C | −2.585 | 2.928 | 0.120 |
| C | −3.441 | 1.775 | −0.651 | C | −3.548 | 1.889 | 0.111 |
| C | −2.773 | 0.588 | −0.151 | C | −2.811 | 0.618 | 0.037 |
| C | −1.231 | 2.228 | −0.199 | C | −1.263 | 2.277 | 0.044 |
| C | 0.000 | 4.374 | 0.000 | C | 0.000 | 4.418 | 0.000 |
| C | −0.637 | 5.086 | 1.026 | C | 0.109 | 5.133 | 1.201 |
| C | −0.630 | 6.481 | 1.031 | C | 0.109 | 6.529 | 1.203 |
| C | 0.000 | 7.182 | −0.001 | C | −0.001 | 7.230 | 0.000 |
| C | 0.630 | 6.481 | −1.032 | C | −0.110 | 6.529 | −1.203 |
| C | 0.636 | 5.086 | −1.027 | C | −0.110 | 5.133 | −1.201 |
| C | −4.852 | −0.795 | 0.171 | C | −4.856 | −0.843 | −0.007 |
| C | −5.505 | −0.249 | 1.287 | C | −5.558 | −1.046 | 1.190 |
| C | −6.890 | −0.345 | 1.415 | C | −6.941 | −1.236 | 1.182 |
| C | −7.645 | −0.975 | 0.423 | C | −7.642 | −1.230 | −0.027 |
| C | −7.006 | −1.518 | −0.694 | C | −6.952 | −1.035 | −1.226 |
| C | −5.618 | −1.433 | −0.816 | C | −5.569 | −0.844 | −1.215 |
| C | 4.852 | −0.794 | −0.170 | C | 4.856 | −0.843 | 0.007 |
| C | 5.618 | −1.432 | 0.817 | C | 5.569 | −0.844 | 1.215 |
| C | 7.005 | −1.517 | 0.696 | C | 6.952 | −1.034 | 1.226 |
| C | 7.645 | −0.975 | −0.422 | C | 7.642 | −1.229 | 0.027 |
| C | 6.891 | −0.344 | −1.414 | C | 6.941 | −1.235 | −1.182 |
| C | 5.505 | −0.249 | −1.286 | C | 5.558 | −1.046 | −1.190 |
| C | −1.708 | −5.335 | 0.758 | C | −1.830 | −5.435 | −0.217 |
| C | −4.135 | −3.868 | 0.857 | C | −4.227 | −3.930 | −0.189 |
| C | 2.803 | 4.070 | 1.221 | C | 3.004 | 4.265 | −0.205 |
| C | 4.745 | 2.014 | 1.128 | C | 4.915 | 2.195 | −0.184 |
| C | 4.135 | −3.867 | −0.857 | C | 4.227 | −3.930 | 0.189 |
| C | 1.708 | −5.335 | −0.759 | C | 1.831 | −5.435 | 0.217 |
| C | −2.804 | 4.070 | −1.222 | C | −3.004 | 4.265 | 0.206 |
| C | −4.746 | 2.013 | −1.128 | C | −4.915 | 2.194 | 0.184 |
| C | −5.061 | 3.266 | −1.629 | C | −5.313 | 3.526 | 0.263 |
| C | −4.093 | 4.287 | −1.680 | C | −4.365 | 4.553 | 0.275 |
| C | 4.093 | 4.288 | 1.680 | C | 4.364 | 4.553 | −0.275 |
| C | 5.060 | 3.267 | 1.629 | C | 5.313 | 3.527 | −0.264 |
| C | 4.095 | −5.216 | −1.192 | C | 4.255 | −5.320 | 0.279 |
| C | 2.894 | −5.947 | −1.142 | C | 3.068 | −6.067 | 0.299 |
| C | −2.893 | −5.948 | 1.141 | C | −3.067 | −6.067 | −0.299 |
| C | −4.094 | −5.217 | 1.191 | C | −4.254 | −5.320 | −0.279 |
| H | −1.134 | 4.540 | 1.823 | H | 0.194 | 4.586 | 2.137 |
| H | −1.120 | 7.021 | 1.837 | H | 0.194 | 7.068 | 2.143 |
| H | 1.119 | 7.021 | −1.838 | H | −0.195 | 7.068 | −2.143 |
| H | 1.133 | 4.539 | −1.823 | H | −0.195 | 4.586 | −2.137 |
| H | −4.918 | 0.249 | 2.054 | H | −5.012 | −1.051 | 2.129 |
| H | −7.381 | 0.077 | 2.288 | H | −7.471 | −1.388 | 2.119 |
| H | −7.587 | −2.007 | −1.471 | H | −7.490 | −1.032 | −2.170 |
| H | −5.120 | −1.855 | −1.684 | H | −5.033 | −0.692 | −2.147 |
| H | 5.119 | −1.855 | 1.685 | H | 5.033 | −0.691 | 2.147 |
| H | 7.586 | −2.006 | 1.473 | H | 7.490 | −1.031 | 2.170 |
| H | 7.382 | 0.077 | −2.287 | H | 7.471 | −1.388 | −2.118 |
| H | 4.919 | 0.249 | −2.053 | H | 5.013 | −1.051 | −2.129 |
| H | −8.725 | −1.043 | 0.520 | H | −8.719 | −1.378 | −0.034 |
| H | 0.000 | 8.269 | −0.001 | H | −0.001 | 8.317 | 0.000 |
| H | 8.726 | −1.043 | −0.518 | H | 8.719 | −1.377 | 0.034 |
| H | −0.774 | −5.889 | 0.761 | H | −0.922 | −6.024 | −0.265 |
| H | −5.070 | −3.326 | 0.921 | H | −5.156 | −3.376 | −0.192 |
| H | 2.072 | 4.864 | 1.292 | H | 2.300 | 5.082 | −0.224 |
| H | 5.496 | 1.236 | 1.124 | H | 5.668 | 1.422 | −0.184 |
| H | 5.070 | −3.325 | −0.921 | H | 5.157 | −3.376 | 0.192 |
| H | 0.775 | −5.888 | −0.762 | H | 0.923 | −6.024 | 0.265 |
| H | −2.073 | 4.863 | −1.292 | H | −2.301 | 5.082 | 0.224 |
| H | −5.497 | 1.236 | −1.123 | H | −5.668 | 1.421 | 0.183 |
| H | −6.064 | 3.456 | −2.001 | H | −6.373 | 3.763 | 0.320 |
| H | −4.357 | 5.258 | −2.092 | H | −4.686 | 5.589 | 0.342 |
| H | 4.356 | 5.259 | 2.091 | H | 4.685 | 5.590 | −0.342 |
| H | 6.063 | 3.457 | 2.001 | H | 6.372 | 3.764 | −0.321 |
| H | 5.009 | −5.712 | −1.506 | H | 5.212 | −5.830 | 0.344 |
| H | 2.892 | −6.997 | −1.422 | H | 3.113 | −7.149 | 0.384 |
| H | −2.891 | −6.998 | 1.421 | H | −3.112 | −7.149 | −0.384 |
| H | −5.008 | −5.713 | 1.505 | H | −5.211 | −5.831 | −0.344 |
